# The temporal dynamics of dissociation: protocol for an ecological momentary assessment and laboratory study in a transdiagnostic sample

**DOI:** 10.1186/s40359-023-01209-z

**Published:** 2023-06-07

**Authors:** Johannes B. Heekerens, James J. Gross, Sylvia D. Kreibig, Katja Wingenfeld, Stefan Roepke

**Affiliations:** 1 Department of Psychiatry and Neurosciences, Charité – Universitätsmedizin Berlin, 12203 Berlin, Germany; 2 Department of Psychology, Stanford University, Stanford, CA 94305 USA

**Keywords:** Dissociation, Depersonalization/derealization Amnesia, Affect, Temporal dynamics, Trauma, Borderline personality disorder, Posttraumatic stress disorder, Dissociative disorder, Psychophysiology

## Abstract

**Background:**

Dissociation is a ubiquitous clinical phenomenon. Dissociative disorders (DD) are primarily characterized by dissociation, and dissociative states are also a criterion for borderline personality disorder (BPD) and the dissociative subtype of post-traumatic stress disorder (PTSD). Dissociative reactions (e.g., depersonalization/derealization or gaps in awareness/memory) across diagnostic categories are believed to be affect contingent and theorized to serve affect regulation functions. What is not clear, however, is how self-reported affect and physiological reactivity unfold within dissociative episodes. To address this issue, the present project aims to investigate the hypothesis (1) whether self-reported distress (as indicated by arousal, e.g., feeling tense/agitated, and/or valence, e.g., feeling discontent/unwell) and physiological reactivity increase before dissociative episodes and (2) whether self-reported distress and physiological reactivity decrease during and after dissociative episodes in a transdiagnostic sample of patients with DD, BPD, and/or PTSD.

**Methods:**

We will use a smartphone application to assess affect and dissociation 12 times per day over the course of one week in everyday life. During this time, heart and respiratory rates will be remotely monitored. Afterwards, participants will report affect and dissociative states eight times in the laboratory before, during, and after the Trier Social Stress Test. During the laboratory task, we will continuously record heart rate, electrodermal activity, and respiratory rate, as well as measure blood pressure and take salivary samples to determine cortisol levels. Our hypotheses will be tested using multilevel structural equation models. Power analyses determined a sample size of 85.

**Discussion:**

The project will test key predictions of a transdiagnostic model of dissociation based on the idea that dissociative reactions are affect contingent and serve affect regulation functions. This project will not include non-clinical control participants. In addition, the assessment of dissociation is limited to pathological phenomena.

**Supplementary Information:**

The online version contains supplementary material available at 10.1186/s40359-023-01209-z.

## Background

Dissociation is a ubiquitous clinical phenomenon defined as “disruption of and/or discontinuity in the normal integration of consciousness, memory, identity, emotion, perception, body representation, motor control, and behavior” [1], p. 329; also see World Health Organization, 86]. Dissociative disorders (DD), such as depersonalization/derealization disorder, are primarily characterized by dissociation, and dissociative states are also a criterion for borderline personality disorder (BPD) and the dissociative subtype of post-traumatic stress disorder (PTSD; [1]). High levels of dissociation have also been demonstrated in eating disorders, substance use disorders, and affective disorders (see [46] for a meta-analysis).

### A transdiagnostic model of the temporal dynamics of dissociation

There has been a robust interest in dissociation over the past decades [18], however, one impediment to further progress on treatments aimed at reducing dissociation is an incomplete understanding of the temporal dynamics of dissociation [72].

Current neurobiological and clinical models posit that dissociation functions to automatically and nonvoluntarily regulate affect following perceived threat [1, 42, 64, 80]. We use affect as the “umbrella term for states that involve relatively quick good-for-me bad-for-me discriminations” [24], p. 3]. Affective states include among other things stress responses in situations that exceed an individual’s ability to cope and negative emotions such as anxiety or depression. Distress occurs when stress responses and/or negative emotions are severe, prolonged, or both. A current meta-analytic review describes two main functions of dissociation within affect regulation, namely non-deliberate avoidance and over-control of distressing situations and related reactions [14]. Dissociation is theorized to occur on a continuum that ranges from milder forms with no or minimal interference with daily functioning (e.g., daydreaming, absorption) to pathologically pervasive forms that can significantly interfere with daily functioning (e.g., depersonalization/derealization, amnesia, stupor, [58]). Because our aim is to further progress on clinical models of dissociation in order to improve treatments, we focus on pathological forms of dissociation in this project, which are frequently observed in trauma-related disorders (e.g., DD, BPD, PTSD).

Trauma models explain that dissociation is one of several possible protective and evolutionarily beneficial responses in extremely dangerous situations, and that the dissociative reaction pattern can repeat itself after traumatic threats when associated threat networks are activated ([51, 66], see [29, 42] for a discussion of the neurological basis of dissociation). Importantly, it is believed that threat networks can become detached from contextual cues related to traumatic experiences, and dissociation can occur as an automatic response to a variety of perceived threats and daily stressors, not only those that are trauma related [49]. Once threat networks have become sensitized in this way, dissociation automatically appears as affective states reach a certain quality, for example, self-reported distress accompanied by increased levels of sympathetic nervous system activity. During a dissociation, increased parasympathetic activity and increased negative feedback at the hypothalamus and pituitary (HPA) axis have been theorized to gradually shutdown physiological reactivity [51, 65, 66]. At the same time, dissociative states may function as automatic affect regulation strategies through nonvoluntarily and quickly deflecting attention away from internal and external perceived threats, altering the cognitive processing of threat-related material by a disruption of the normal integration of thoughts, sensations, and perceptions in a way that prevents threatening information from being further processed, as well as influencing appraisal processes by disrupting the development of mental representations of distressing stimuli and sustaining automatic and rigid threat appraisals ([25], see [14, 64] for a discussion). In consequence, self-reported distress should decrease in the short-term, which may then reinforce the dissociative response pattern [32]. Paradoxically, the automatic regulation of affect through avoidance strategies might come at the cost of heightened distress in the long-term. For one thing, it is well-documented that affect avoidance increases the future duration, intensity, and distressing quality of affective experiences [26]. In addition, dissociation might not allow to deploy more adaptive regulation strategies before (e.g., problem-solving), during (e.g., mindfulness), and after (e.g., reappraisal) confronting stimuli perceived as threatening.

### Research status and gap

While available evidence informs some predictions made by a transdiagnostic model of the temporal dynamics of dissociation, other predictions remain to be tested. Existing studies show that most individuals fulfill criteria of disorders associated with past trauma and maltreatment (e.g., DD, BPD, PTSD; [46, 71]). In addition, a robust body of evidence links retrospectively assessed childhood abuse and neglect to affect contingent dissociation later in life ([59], see [81] for a meta-analysis). Many of the patients with dissociative symptoms also report high levels of distress [1, 8]. Studies using multiple assessments per day report positive within-person associations between dissociation and self-reported unpleasant, inner tension (indicating distress) in patients with BPD or PTSD (but not non-clinical controls), suggesting that dissociation is strongest when distress is increased [67, 75]. One study shows that increased self-reported arousal (feeling tense as opposed to calm; one operationalization of distress) precedes dissociation in patients with BPD (but not patients with depression), and that self-reported valence (feeling unpleasant as opposed to pleasant; another operationalization of distress) improves for some patients shortly after a dissociation [30]. However, the study has several methodological limitations and current evidence is insufficient to conclude that distress increases prior to and decreases during dissociation across diagnostic categories. In addition, laboratory studies have shown increased dissociation during or shortly after exposure to various stressors such as personalized stressful narratives [15], arousal induced by the hyperventilation provocation test [53], panic induced by carbon dioxide inhalation [62], psychosocial stress [23, 48, 83], and trauma reminders ([16, 88], see [39, 45] for reviews). Changes in physiological parameters that serve as markers for autonomous nervous system activity during dissociation have also been investigated, but current evidence is mixed (see [7, 63] for reviews). For example, laboratory studies find increases, decreases, or no changes in cardiovascular measures during dissociation. Results, however, are mostly based on samples well below *N* = 30. Two studies with larger samples that measures heart rate variability metrics heart rate variability after a dissociation response demonstrate increased respiratory sinus arrhythmia (RSA) in patients with depersonalization disorder after a biofeedback training [68] and higher low-frequency/high-frequency (LF/HF) ratio (but no change in RSA) in patients with PTSD after the TSST [57]. Results from another study with N = 71 patients with PTSD suggest increased RSA and increased non-specific skin conductance response (NS.SCR), as well as a nonlinear relation (inverted U-shape) with heart rate during dissociation induced by a trauma script paradigm [16]. One review found lower salivary cortisol levels in patients with PTSD and dissociative symptoms compared to healthy controls after stress exposure in the laboratory [10]. One limitation of these experiments it that physiological and dissociative states are either assessed only at baseline or before and after but not during stress paradigms, which makes it difficult to reliably capture change dynamics. Therefore, a key prediction of trauma models, the specific temporal physiological profile of a dissociation, remains largely untested. This gap has also been pointed out in a recent review [14]. In addition, very few studies have adopted a transdiagnostic approach to investigate shared temporal antecedents and consequences of dissociative responses between disorders. Studies that focus on distinct diagnostic groups may reveal aspects of dissociation specific to these groups, but are often limited as they do not investigate common processes underpinning dissociative reactions. Learning about the temporal dynamics of dissociation in a transdiagnostic sample would help to fill these gaps, and further increase our understanding of whether and at what intervals distress increases prior to dissociation, how quickly dissociation appears, and whether dissociation is effective in reducing distress.

### Moderators of the link between affect and dissociation

As explained above, we assume increases in distress to precede dissociation across diagnostic groups provided that the patient reports a general pattern of dissociative reactions. The size of this effect, however, should vary between patients. Our literature review suggests at least three potential moderatos. First, we expect patients who report more exposure to past trauma, the single most important etiological factor linked to dissociation, to report a stronger link between distress and dissociation because with higher exposure to past trauma fear networks are more likely to become detached from contextual cues [49, 66]. Second, patients who report more coping capabilities other than dissociation (e.g., emotion regulation, social support) should report a weaker link between distress and dissociation because stress and/or negative emotions should be effectively modulated more often [14].

## Methods and design

### Aim of the present study

This investigation is unified by the overarching aim of further developing a concise and reliable model of how dissociative symptoms unfold and are maintained. To achieve this goal, we examine how self-reported affect and physiological reactivity unfold within dissociative episodes in an adequately sized transdiagnostic sample of patients with dissociative symptoms (DD, BPD, PTSD. The repeated assessment of dissociation includes experiences of depersonalization/derealization and gaps in awareness/memory, which are at the core of current definitions of dissociation [1, 86] and are frequently reported in patient samples (e.g., [12, 38, 76]). We do not assess stupor or fugue because theses can better be assessed retrospectively and/or using behavioral observations.

**Fig. 1:**
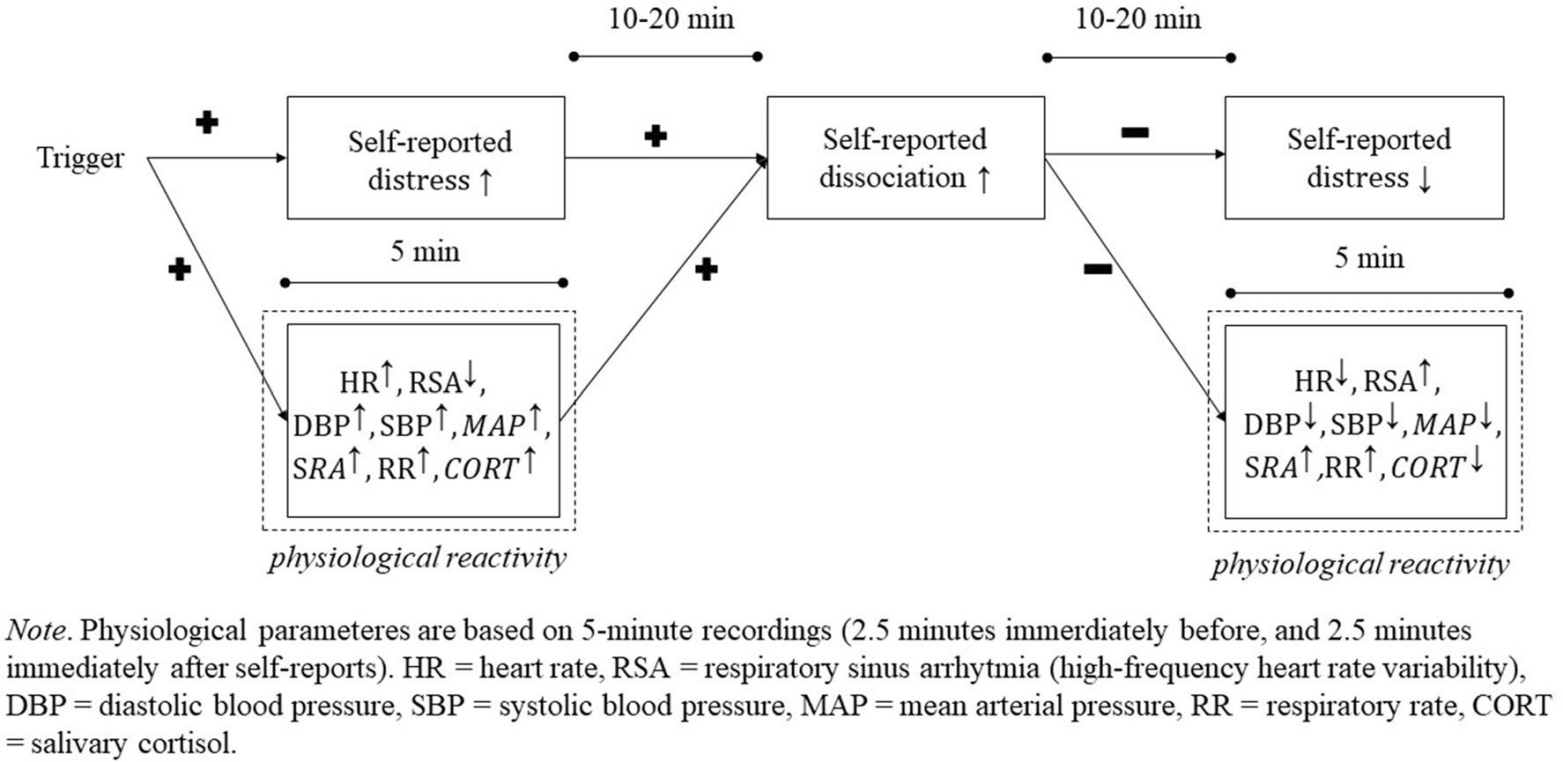
Expected temporal relations between affect, dissociation, and physiological reactivity. *Note*. Physiological parameters are based on 5-min recordings (2.5 min immediately before, and 2.5 min immediately after self-reports). *HR* heart rate, *RSA* respiratory sinus arrhythmia (high-frequency heart rate variability), *DBP* diastolic blood pressure, *SBP* systolic blood pressure, *MAP* mean arterial pressure, *RR* respiratory rate, *CORT* salivary cortisol

The present study will assess individuals both during their everyday life and in the laboratory using similar measures and statistical models. Figure 1 summarizes how we expect self-reported affect and physiological reactivity to unfold within dissociative episodes. Although past research has demonstrated dissociation in response to external stressful triggers, and some studies have investigated self-reported affect and physiological parameters before and after (but not during) dissociations, several predictions of the model depicted in Fig. 1 require further testing. Specifically, we hypothesize:

1. First, we expect that increases in self-reported distress (as indicated by arousal, i.e., feeling tense/agitated, and/or valence, i.e., feeling discontent/unwell) precede dissociative reactions both in everyday life and during a stress induction in the laboratory.^1^ During and after a dissociation, we expect self-reported distress to decrease (as indicated by arousal, i.e., feeling relaxed/calm, and/or valence, i.e., feeling content/well).
2. Second, we expect that increases in physiological reactivity (as indicated by increased heart rate, decreased respiratory sinus arrhythmia, increased systolic and diastolic blood pressure, increased electrodermal activity, and increased respiratory rate) precede dissociative reactions both in everyday life and during a stress induction in the laboratory. During and after a dissociation, we expect decreased physiological reactivity (as indicated by decreased heart rate, increased respiratory sinus arrhythmia, decreased systolic and diastolic blood pressure, decreased electrodermal activity, and decreased respiratory rate), as well as increased negative feedback at the HPA axis (as indicated by a decreased salivary cortisol levels).
3. Third, we expect these relations to be larger among patients who report more past trauma and maltreatment, and in patients who report fewer coping capabilities other than dissociation (as indicated by higher baseline dissociation, less adaptive and/or more maladaptive emotion regulation, less social support).

For all our hypotheses, we will investigate potential differences between diagnostic groups (BPD, PTSD, DD) and baseline psychopathology.

### Inclusion and exclusion criteria

The sample will comprise patients with dissociative symptoms. Participants will be at least 18 years old. Participants will be included if they meet DSM-5 criteria for DD, BPD, and/or PTSD. To ensure a sufficient degree of dissociation during our study, we will only include participants whose sum of scores is at least 20 on the Dissociative Symptom Scale or whose sum of scores on any of the depersonalization/derealization or gaps in awareness/memory subscales of the Brief Version of the Dissociative Symptom Scale is at least five [13, 47]. Participants will be excluded if they meet DSM-5 criteria for bipolar disorder, any psychotic disorder, a severe major depressive episode (8 or 9 symptoms present), anorexia nervosa, severe alcohol use disorder (past 3 months), or any substance use disorder (at least moderate in the past 3 months). Patients taking psychotropic medication will not be excluded but medication must have remained stable for at least two weeks before the study and during the assessment period. Medication type and dose will be assessed and controlled for in statistical analyses. We do not plan to include a non-clinical sample because the forms of dissociation investigated in this project rarely occur in such samples and floor effects seem likely that would complicate group comparisons [30].

### Procedures

**Fig. 2:**
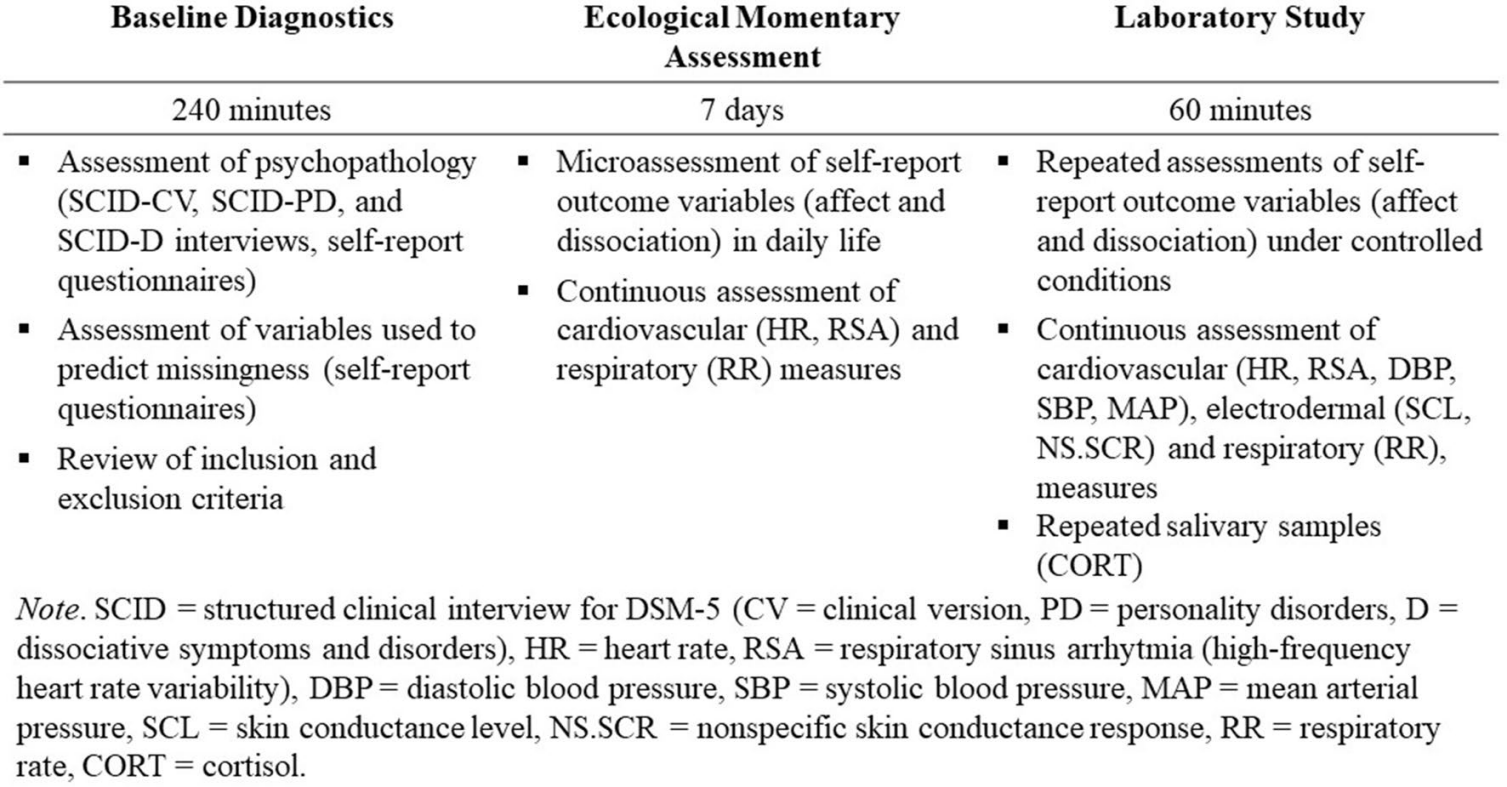
Data collection in N = 85 patients with dissociative symptoms. *Note*. *SCID* structured clinical interview for DSM-5 (*CV* clinical version, *PD* personality disorders, *D* dissociative symptoms and disorders), *HR* heart rate, *RSA* respiratory sinus arrhythmia (high-frequency heart rate variability), *DBP* diastolic blood pressure, *SBP* systolic blood pressure, *MAP* mean arterial pressure, *SCL* skin conductance level, *NS.SCR* nonspecific skin conductance response, *RR* respiratory rate, *CORT* cortisol

Participants will be recruited at the Department of Psychiatry and Neurosciences at Charité – Universitätsmedizin Berlin and through social media advertisement. The ecological momentary assessment will take place during everyday life and not during inpatient treatment. Figure 2 displays the data that will be collected.

#### Baseline diagnostics

All participants will be interviewed using the German versions of the Structured Clinical Inverviews for DSM-5 Clinical Version (SCID-5-CV; [4]), Personality Disorders (SCID-5-PD, [3]), and Dissociative Symptoms and Disorders (SCID-5-D, [73]) to diagnose DD, BPD, and PTSD. We will use the SCID-5-CV to determine presence or absence of current (major) depressive disorder, lifetime bipolar disorder, and any lifetime psychotic disorder.

The following self-report questionnaires will be administered online or in the laboratory using a computer: We will administer the German version of the Dissociative Experience Scale (DES; [22]) and the German version of the Dissociative Symptoms Scale (DSS [12]); to assess baseline dissociation; the German version of the Difficulties in Emotion Regulation Short Form (DERS-SF; [27, 31]) to assess deficits in emotion regulation using 18 items; the German version of the Process Model of Emotion Regulation Questionnaire (PMERQ; [54]) to assess individual differences in emotion regulation using 45 items; subscales of the German version of the Childhood Trauma Questionnaire (CTQ [84]); will be used to retrospectively assess self-reported childhood trauma using 25 items; a subscale of the German version of the Posttraumatic Diagnostic Scale for DSM-5 (PDS-5; [85]) to assess self-reported reexperiencing and avoidance of any trauma-related memories using seven items; the German version of the Patient Health Questionnaire-8 (PHQ-8; [40, 44]) to assess the severity of depressive symptoms using eight items; the German version of the Personality Inventory for DSM-5, Brief Form Plus (PID5BF+; [33]) to assess self-reported psychopathological personality trait facets using 36 items; the German 10 Item Big Five Inventory (BFI-10; [61]); and five items assessing typical phone use (based on [37]).

In addition, we will assess the following demographic and health variables as control variables: age, gender, marital status, ethnicity, highest general education degree, employment situation, night shifts, smoker (pack years), height, weight, somatic diseases, current use of psychotropic or other drugs including needs medication. Biological women will be asked to indicate pregnancy, contraceptive use, menopause status, uterus and/or ovaries removal, menstruation regularity, and menstruation cycle.

Full lists of clinician-administered and self-report questionnaires are available in the online supplements.

#### Experience sampling

After baseline diagnostics, participants will download an app (“m-path”) to their smartphones or receive a smartphone including the app [52]. Participants will be instructed to go on with their daily activities and respond to several questions when prompted by a beep. The app will be programmed to beep once every day at 9:00 AM and 9:00 PM, as well as 12 times every day between 10:00 AM and 9:00 PM for seven consecutive days. At 9:00 AM and 9:00 PM we will assess contextual information (see assessment of context information section). Between 10:00 AM and 9:00 PM we will assess affect and dissociation (see assessment of self-reported affect and dissociative states section). Four consecutive prompts will be distributed throughout the day within three random 60-min intervals starting at random times in the morning, afternoon, and evening (variable timing schedule). The time between two consecutive prompts is 15 min (based on preliminary results reported by Heekerens et al. [30], see [19] for a discussion). If the first beep occurs at 9:00 AM, the second beep will follow at 9:15 AM, the third at 9:30 AM, and the fourth at 9:45 AM. The fifth beep may occur at 1:15 PM, followed by the sixth beep at 1:30 PM, and so on. Prompts will be set to expire after 5 min to ensure that the time between two consecutive answers is between 10 and 20 min. To ensure uniform time intervals, prompts are triggered at the full hour, half hour, or quarter hour. This approach creates a grid with 48 possible time points for prompts each day, 12 of which are realized. The variable times between consecutive prompts (e.g., 9:45 AM and 1:15 PM) can be appropriately dealt with in our statistical analysis by defining missing values for the 36 unrealized time points each day [2]. The advantage of this approach is that it helps to realize a dense sampling plan that generalizes across the day while reducing participant burden. Dense sampling helps to capture dynamical features of affect and ensures a meaningful number of dissociative episodes. Responses will be time-stamped by the software.

#### Laboratory study

**Fig. 3:**
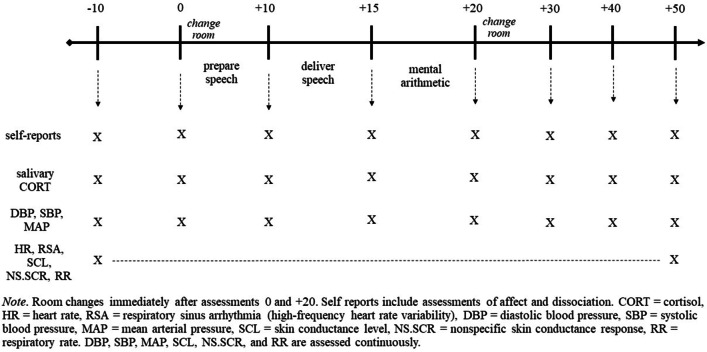
Self-report and physiological assessments during the Trier Social Stress Test (TSST) in the laboratory. *Note*. Self reports include assessments of affect and dissociation. *CORT* cortisol, *HR* heart rate, *RSA* respiratory sinus arrhythmia (high-frequency heart rate variability), *DBP* diastolic blood pressure, *SBP* systolic blood pressure, *MAP* mean arterial pressure, *SCL* skin conductance level, *NS.SCR* nonspecific skin conductance response, *RR* respiratory rate. DBP, SBP, MAP, and RR are assessed continuously

After the experience sampling, patients will participate in the Trier Social Stress Test (TSST; based on [34] and following the guidelines by [43]). The TSST has three parts. In the first part, participants are asked to prepare a speech for 5 min. In the second part, participants deliver the speech for 5 min in front of two judges (one male and one female), who are trained to respond in a discouraging way and take long pauses. In the third part, participants are asked to perform mental arithmetic (1022-13) in front of the judges for 5 min. Participants will be asked to indicate levels of dissociative and affect, as well as take a salivary sample and blood pressure measure after each part of the TSST (Fig. 3). Heart rate, blood pressure, electrodermal activity, and respiratory rate will be measured continuously. We will carefully control environmental factors that can influence cardiovascular and cortisol results (see the Additional file 1, and Additional file 3 for details).

### Assessment of self-reported affective and dissociative states

Participants will indicate their current affective and dissociative states both in everyday life and during the laboratory task. Constructs will be assessed using at least two items (as recommended by [11, 20]. Single items are problematic because state-specific components of the construct cannot be separated from measurement error.

Momentary affective states will be assessed using items from a validated German measure specifically designed to reliably capture within-person variability [82]. The measure is based on the Multidimensional Mood Questionnaire (MDMQ, [74]) that assesses basic diffuse affect dimensions. In this study, participants will be asked to indicate their levels of arousal using two bipolar items (“relaxed-tense”/“entspannt-angespannt” and “agitated-calm”/“unruhig-ruhig”) and valence using two bipolar items (“content-discontent”/“zufrieden-unzufrieden” and “unwell-well”/“unwohl-wohl”). The items use a slider from the starting position 0 to a maximum of 6. A recent study found good within-person reliabilities of the arousal and valence subscales in a mixed sample of patients with BPD and anxiety disorders (McDonald’s omega = 0.86 and 0.88, respectively; [36]).

Next, participants will be asked to indicate their level of dissociation. We will administer three subscales of the Dissociative Symptoms Scale Brief Form (DSS-B; [47]). The scale was originally designed to retrospectively capture clinically relevant dissociative symptoms of moderate intensity in the past two weeks. The DSS-B has recently been translated to German by Nikolaus Kleindienst (personal communication, April 04, 2023). Items of the DSS-B have demonstrated sufficient within-person variability in an experience sampling study using 4-h periods [12]. For this study, we adapted the DSS-B subscales to capture experiences “in the moment”. Participants will be asked to answer two items indicating momentary depersonalization/derealization (“At the moment, things around me seem strange or unreal.” and “…, I feel like I am in a movie—like nothing that is happening is real.”) and two items indicating gaps in awareness/memory (“At the moment, I realize I am not paying attention to what is going on around me.” and “At the moment, I am so focused on something going on in my mind that I lose track of what is happening around me.”) using a scale ranging from 0 (*not at all)* to 4 (*very*). See the Additional file 4 for details.

#### Assessment of context information

The following variables will be used as controls. At the start of each day during the experience sampling, participants will fill out a single question (“Last night, I had a problem with my sleep”) assessing daily sleep disturbance using a scale ranging from 0 (*not at all*) to 4 (*very*). Sleep disturbance has been shown to impact daytime affect (see ten [78] for a review). During the day, participants will be asked about situational experiences after each block of four prompts using each one item for three subscales of the German Personality Dynamics Diary [87]. We chose items with the largest within-person factor loadings (social stress: “In the past hour, I was ignored or rejected by others”, positive event: “In the past hour, I had a good time with others (e.g., interesting or funny conversations)”, workload: “In the past hour, I was under high pressure to succeed while getting done with my tasks”). The items use a simplified yes/no answer format to reduce participant burden. In addition, participants will indicate whether they exercised in the past hour (yes/no). Finally, at the end of the day, participants will respond to three questions assessing daily levels of distress (stress: “Today, I felt stressed”, anxiety: “Today, I felt fearful”, depression: “Today, I felt worthless”) and one item assessing daily social isolation (“Today, I felt left out”) using a scale ranging from 0 (*not at all*) to 4 (*very*), as well as one question asking about daily consumption of illicit drugs (yes/no).

#### Cardiovascular and respiratory assessments

We will use heart rate (HR) and respiratory rate (RR) to indicate combined sympathetic and parasympathetic activity, and a heart rate variability metric (respiratory sinus arrhythmia, RSA) to indicate parasympathetic activity. Electrocardiogram (ECG) recordings will be sampled both in everyday life and in the laboratory with a frequency of 128 Hz using portable three-lead recorders developed by VivaLink, Campbell, USA (model: VV330). Traditional sources suggest a sampling rate of at least 250 Hz to ensure satisfactory estimation of R-peak location and subsequent calculations of heart rate variability metrics [5, 77]). However, current evidence suggests that any bias at sampling rates down to 125 Hz may be negligible [21]. R-wave fiducial points will be mathematically refined prior to calculation of heart rate variability metrics, which should help to reduce potential bias due to low sampling frequency (R-peak interpolation; [77]). Data will be cleaned in two steps. First, we will use Kubios software (www.kubios.com) to automatically detect artifacts from a time series consisting of differences between successive RR intervals. Second, we will visually inspect the automatically cleaned ECG signal and manually remove all remaining artifacts. Afterwards, Kubios software will be used to detect R-waves in the ECG to calculated consecutive R–R intervals and quantify the RSA parameter by calculating the absolute power (in milliseconds squared, ms^2^) of the high-frequency or respiratory band (0.15–0.40 Hz), reflecting parasympathetic (or vagal) influence on the heart. HR (in beats per minute, bpm) will be measured in the range of 40–300 bpm. RR (in breaths per minute, brpm) will be measured in the range of 5–35 brpm. The respiratory signal will be derived based on the ECG. Following conventions, we will use 5-min recordings covering the time immediately before participants submit self-reports to calculate mean HR, mean RR, and RSA [77]. The VivaLink ECG monitor also assesses movement (5 Hz 3-axis accelerometer) and peripheral (skin) temperature, and these data will be used to facilitate the interpretability of our results.

In the laboratory study, we will additionally monitor blood pressure using a blood pressure cuff developed by iHealth, Paris, France (model: Neo BP5S). The cuff will be placed on the participant’s nondominant upper arm (brachial artery) at the height of the heart (as recommended by [6]. Blood pressure is measured continuously in units of millimeters of mercury (mmHg), and we will compute the highest blood pressure seen at systole (SBP, range: 60–260 mmHg), the lowest seen in diastole (DBP, 40–199 mmHg), and the mean arterial pressure (1/3*SBP + 2/3*DBP).

#### Electrodermal assessment

In the laboratory, we will use skin conductance levels (SCL) and frequency of nonspecific skin conductance responses (NS.SCR) as additional indicators of sympathetic activity (as recommended by the Society for Psychophysiological Research; [9]). During the ecological momentary assessment phase and laboratory study, a raw electrodermal activity signal will be sampled continuously using a textile band worn on the dominant wrist (based on) with electrodes placed on the skin developed by Empatica Inc. (model: EmbracePlus; [55, 56]). Although some studies find that SCL and NS.SCR measures based on the recordings of wrist-worn devices only moderately correlate with measures obtained from traditional palmar devices, wrist-worn devices have been shown to successfully detect intra-individual differences in arousal levels across a variety of contexts including clinical applications ([35], see [17] for a review). Skin conductance will be measured in units of microsiemens (μS) and in the range of 2–100 μS. The sampling rate is 4 Hz, meeting minimum requirements [70]. Data from artifacts (e.g., caused by pressure on the device) will be corrected using EDA explorer (https://eda-explorer.media.mit.edu/) and visual inspection of the raw electrodermal signal [9]. We will use 5-min clean electrodermal recordings covering the time immediately before participants submit self-reports to calculate mean SCL and frequency of NS.SCR. EDA explorer will be used to detect peaks in 5-s periods of the clean electrodermal 5-min signal. The NS.SCR will be calculated as the number of skin conductance increases from a zero-slope baseline exceeding 0.025 μS per minute. The EmbracePlus device also assesses acceleration and rotation (actigraphy) and blood volume pulse (BVP) by photoplethysmography (PPG) at sampling rates of 64 Hz, as well as peripheral (skin) temperature at sampling rates of 1 Hz. We will use these metrics to aid interpretation.

#### Cortisol assessment

We will obtain salivary cortisol to measure activation of the HPA axis during TSST in the laboratory study. As shown in Fig. 3, saliva samples will be collected after arrival at approximately 10 min and immediately before preparing the speech (pre-TSST), immediately before delivering the speech, immediately before the mental arithmetic task, and immediately post, 10, 20, and 35 min after the mental arithmetic task (post-TSST). We will use cellulose pledges developed by Sarstedt AG (Salivette) that can be placed in plastic carriers designed for low-temperature storage. Samples will be immediately frozen and stored at − 80 °C until biochemical analysis, which will be performed in the Neurobiology Laboratory of the Department of Psychiatry and Neurosciences, Charité – Universitätsmedizin Berlin, Germany.

#### Procedures to enhance compliance

We will seek to enhance compliance during experience sampling by thoroughly training staff and participants in the study procedures, by implementing a training session for participants, by giving clear instructions, and by using a remuneration schedule (as recommended by [79]). Each participant will meet with the investigator or a qualified research assistant at the beginning of the study to undergo a supervised “practice” survey with the opportunity to ask questions. Participants will receive extra financial incentive for high levels of compliance (> 80%) and will be able to view their progress in the study.

### Statistical analyses

Preliminary analyses will be performed using R [60] and hypotheses will be tested using multi-level models in Mplus [50]. All models will use latent variables measured by two observed variables for any self-reports, which will help to separate true systematic variance from unsystematic variance due to measurement error to avoid estimation bias in model parameters. For dimensional self-report constructs, we will report multilevel reliability coefficient omega values to indicate the reliability of the overall composite score, as well as on the within-level and between-level (as recommended by [41]). In addition, we will report average within-person standard deviations and intraclass correlations of all within-person measures. We will use a Bayes algorithm without distribution assumptions. We will use the Mplus default priors and investigate model fits using potential scale reduction factor (cut-off: < 1.10) and careful inspection of trace plots. The models used to test hypotheses in the ecological momentary assessment and laboratory data sets are very similar but not identical as ecological momentary assessment involves more time points.

We will test ecological momentary assessment hypotheses using dynamic structural equation modeling (SEM; [2]) and laboratory study hypotheses using random intercept cross-lagged panel models (RI-CLPM, [28]). Both approaches separate interindividual between-person differences (trait levels) from within-person fluctuations around this value and allow investigation of interindividual differences in autoregressive and cross-lagged associations. The dynamic SEMs will include autocorrelations of order 1 (AR[1]) and cross-lagged associations at the within-person level. The models will allow for person-specific random innovation variances. All models will take varying time intervals between assessments into account by inserting missing data for omitted prompts [2]. Hypothesis tests will be performed in several steps for both data sets. First, we will run separate models relating dissociative states with (a) arousal, (b) valence, and (c) physiological parameters. Physical activity metrics based on accelerometer and rotation data will be included as controls in models including ambulatory physiological variables (e.g., heart rate). When analyzing salivary cortisol, we will control for the phase of menstrual cycle in female participants, age, and other potentially influencing variables. Second, we will include two or more predictor variables (e.g., arousal and valence) in the same model. Third, we will analyze differences between patients by including baseline scores (e.g., difficulties in emotion regulation) as between-level predictors or context variables (e.g., current stressful event) as within-level predictors. Fourth, we will investigate differences between diagnostic groups. Afterwards, sensitivity and additional analyses will be performed (e.g., examining potential influences of medication status, etc.).

#### Handling of missing data

Missing data will be handled within the Bayesian estimation algorithm using multiple imputations [2]. Because the procedure assumes that data are missing at random, we will assess variables to predict missingness (e.g., conscientiousness and items assessing phone use, e.g. “I regularly check my phone even if it does not ring”). We will perform multilevel logistic regression models to examine whether these or other exogenous variables (e.g., general psychopathology) in our data set are related to dichotomous missingness indicators. If they are, we will include them in our models as auxiliary variables (as recommended by [20]).

### Power analyses

Sample size was determined using Monte Carlo simulations (Mplus version 8.8). The models in the simulations match the models we intend to use for our hypothesis tests. As in traditional power analysis, the simulations assume the size of the expected effects and effect variances. Our primary interest is in cross-lagged effects linking self-reported affect and dissociation (hypothesis 1). Power was set to at least 0.80 for these effects and the alpha level was set to 0.05. Other than traditional power analysis, Monte Carlos simulations also make assumptions about effects and variances that are only indirectly related to our hypothesis tests. These effects and variances include fixed effects, random effects variances, and innovations in the RI-CLPMs and dynamic SEMs we will use to test our hypotheses. Code and full results are available at https://osf.io/qwz27/.

#### Experience sampling study

Effect estimates for the experience sampling study simulation dynamic SEMs are based on pilot data from our working group that used dynamic SEM in patients with BPD ([30], see [69] for details). The experience sampling of the current study comprises 84 assessments (12 assessments per day over one week). With *N* = 85, this will result in 7140 data points. We expect an average of 16 missing responses (approx. 80% compliance). For hypothesis 1, we assume that the fixed effect of the temporal relation between arousal (*t* − 1) and subsequent dissociative states (*t*) is 0.15 in our dynamic SEM, which is a conservative estimate based on earlier results (effect in pilot study: 0.25; [30]). We estimate that our model will have a power of 0.98 to detect an effect of 0.15.

#### Laboratory study

Effect estimates for the laboratory study simulation RI-CLPMs are based on pilot data from our working group that investigated the effects of the TSST on dissociative states in patients with BPD and/or PTSD [23]. For hypothesis 1, we assume that the fixed effect between arousal after the first part of the TSST (preparing the speech, *t* − 1) and dissociative states after the second part of the TSST (delivering the speech, *t*) will be 0.30. The estimate is based on an earlier study that reports an effect of 0.25 across everyday life situations [30], and can be considered conservative as the association between arousal and dissociative states is expected to be higher under stress [23]. In a sample of *N* = 85, we estimate the power of our model to detect the effect of 0.30 to be 0.88.

## Supplementary Information

**Additional file 1.** Self-report measures.

**Additional file 2.** Clinician administered interviews.

**Additional file 3.** Laboratory session.

**Additional file 4.** Dissociative states measure.

## Data Availability

The current project has been pre-registered with AsPredicted on 20 April 2023 (https://aspredicted.org/cw6f7.pdf, ID: No. 129440). A protocol for the laboratory procedures is available (https://www.protocols.io/view/tsst-cukywuxw, ID: 75538). After an initial embargo period, pseudonymized individual patient data will be made available (https://doi.org/10.5281/zenodo.7568590). Data usage will be allowed for all purposes (public use file). Researchers who wish to gain access to the data before the end of the embargo period are invited to contact the corresponding author. All statistical scripts will also be made available (https://osf.io/qwz27/).

## Footnotes

1 We expect levels of self-reported distress, physiological reactivity, and levels of dissociation to increase due to anticipation of the stressful speech (before the TSST). The increase should continue during the speech and arithmetic tasks (first and second TSST task). During or shortly after the arithmetic task (second task or recovery period), we expect self-reported distress and physiological reactivity to decrease in those participants who reported increased dissociative states in anticipation (before the TSST) and during the speech (first task).

## Declarations

### Data handling

At baseline, each participant will be assigned an ID, and the same ID will be used throughout the study for all measures. All study computers will be password-protected and kept in locked offices. We will maintain a secure, password-protected study server for data storage. Our local computer networks are secured by mandatory log-in to individual password-protected accounts and will be audited daily for unauthorized access. Monthly backup tapes will be kept in locked, offsite safe deposit boxes. Data with identifiers, such as names or addresses, will be separated from the study data and kept only at the clinical sites and will be password protected. All data analyses will be performed without patient identifiers. All data and procedures will be archived for at least 10 years after publication.

### Patient and public involvement

Patient partners and advocates have not been formally involved in the design of this study. All questions and procedures were designed with the priorities, experiences, and preferences of patients in mind.
