# Supplementary material for: The temporal dynamics of dissociation: protocol for an ecological momentary assessment and laboratory study in a transdiagnostic sample
Source: BMC Psychol. 2023 Jun 7;11:178. doi: 10.1186/s40359-023-01209-z (PMC10245627; doi:10.1186/s40359-023-01209-z)
Supplement: Supplementary file 1 — Additional file 1. Self-report measures. [file 40359_2023_1209_MOESM1_ESM.docx]

This document contains a list of all self-report measures used in the project.

**Baseline Questionnaires**

The following measures were included to describe our sample and as control variables (e.g., Linares et al., 2020).

How old are you? ___ years [age]

German: Wie alt sind Sie? ___ Jahre

To which gender do you assign yourself? [gender]

German: Welchem Geschlecht ordnen Sie sich zu?

1. weiblich (female)
2. männlich (male)
3. nicht-binär/genderqueer (non-binary/genderqueer)
4. kein Geschlecht (no gender)
5. keine Angabe (no answer)
6. Sonstiges (bitte angeben) (open write-in option)

What is your marital status? [marital_status]

Was ist Ihr Familienstand?

1. Ledig (single)
2. In fester Partnerschaft aber nicht verheiratet und lebe mit meinem/meiner Partner/-in zusammen (in a steady partnership but not married and living together with my partner)
3. In fester Partnerschaft aber nicht verheiratet und lebe von meinem/meiner Partner/-in getrennt (in a steady partnership but not married and living separately from my partner)
4. Verheiratet und lebe mit meinem/meiner Ehepartner/-in zusammen (married and living with my spouse)
5. Verheiratet und lebe von meinem/meiner Ehepartner/-in getrennt (married and living separately from my spouse)
6. Geschieden (divorced)
7. Verwitwet (widowed)

Which ethnicity fits for you? [ethnicity]

German: Welche Ethnie passt für Sie?

1. Weiß oder kaukasisch (White or Caucasian)
2. Arabisch oder türkisch (Arab or Turkish)
3. Asiatisch (Asian)
4. Schwarz oder afrodeutsch (Black or Afrogerman)
5. Gemischte Ethnie (mixed ethnicity)
6. keine Angabe (no answer)
7. Sonstige (bitte angeben) (open write-in option)

What is your highest general education degree? [education]

Welchen höchsten allgemeinbildenden Schulabschluss haben Sie?

1. Schüler/-in, besuche eine allgemeinbildende Vollzeitschule (student, attending a full-time general education school)
2. Von der Schule abgegangen ohne Schulabschluss (left school without a diploma)
3. Hauptschulabschluss (Volksschulabschluss) oder gleichwertiger Abschluss (secondary school certificate after 9 years or equivalent qualification)
4. Realschulabschluss (Mittlere Reife) oder gleichwertiger Abschluss (secondary school certificate after 10 years or equivalent qualification)
5. Fachhochschulreife (technical college entrance qualification)
6. Abitur / allgemeine oder fachgebundene Hochschulreife (Gymnasium) (high school diploma / general or subject-specific university entrance qualification)
7. Sonstige (bitte angeben) (other (please specify))

Which employment situation fits for you? [employment]

German: Welche Erwerbssituation passt für Sie?

1. Vollzeiterwerbstätig (full-time employed)
2. Teilzeiterwerbstätig (part-time employed)
3. Gelegentlich oder unregelmäßig beschäftigt (employed occasionally or irregularly)
4. Altersteilzeit (partial retirement)
5. marginally employed, 450-euro-job, mini-job or "one-euro-job" (when receiving citizen's allowance)

German: Geringfügig erwerbstätig, 450-Euro-Job, Minijob oder „Ein-Euro-Job“ (bei Bezug von Bürgergeld)

1. voluntary military service, federal voluntary service or voluntary social year

German: Freiwilliger Wehrdienst, Bundesfreiwilligendienst oder Freiwilliges Soziales Jahr

1. not employed (including: pupils or students not working for money, unemployed, early retirees, pensioners without additional income, leave of absence, maternity leave, parental leave or other)

German: Nicht erwerbstätig (einschließlich: Schüler/-innen oder Studierende, die nicht gegen Geld arbeiten, Arbeitslose, Vorruheständler/-innen, Rentner/-innen ohne Nebenverdienst, Beurlaubung, Mutterschafts-, Erziehungsurlaub, Elternzeit oder sonstige)

Are you working night shifts? [night_shifts]

German: Arbeiten Sie in Nachtschichten?

1. Nein (no)
2. Ja (yes)

Are you smoking? [smoker]

Sind Sie Raucherin bzw. Raucher?

1. Nein (no)
2. Ja (yes)

If yes:

How many cigarettes do you smoke per day? [cigarettes]

How many years have you been smoking? [smoker_years]

German: Falls Ja:

Wie viele Zigaretten rauchen Sie pro Tag?

Seit wie vielen Jahren rauchen Sie?

Größe (height) (cm) [height_cm]

Gewicht (weight) (kg) [weight_kg]

Which is your dominant hand? (In case you are ambidextrous, please select "right").

German: Welches ist Ihre dominante Hand? (Für den Fall, dass Sie beidhändig sind, wählen Sie bitte „rechts“.)

1. right (right-handed)

German: rechts (Rechtshänder)

1. left (left-handed)

links (Linkshänder)

Only for female participants:

Are you currently pregnant? [pregnancy]

German: Sind Sie derzeit schwanger?

1. Nein (no)
2. Ja (yes)

Are you currently using hormonal contraceptives (e.g., contraceptive pill)? [contraceptive]

Nehmen Sie zurzeit hormonelle Verhütungsmittel ein (z. B. die Pille)?

1. Nein (no)
2. Ja (yes)

Menopause (colloquially menopause) is a time when a lot changes hormonally. This may have an influence on the data collected in this study. How do you assess your menopause status? [menopause_status] (based on Harlow et al., 2013; also see Klusman et al., 2022)

German: Die Menopause (umgangssprachlich Wechseljahre) ist eine Zeit, in der sich hormonell viel verändert. Dies kann Einfluss auf die in dieser Studie erhobenen Daten haben. Wie schätzen Sie Ihren Menopause-Status ein?

1. Premenopausal: regular menstrual cycles in the 22- to 35-day range

German: Prämenopause: regelmäßige Menstruationszyklen im Bereich von 22 bis 35 Tagen

1. Early transition: change in cycle length of 7 days or longer in either direction (longer or shorter) from your initial cycle length for at least 2 cycles

German: Frühe Umstellung: Veränderung der Zykluslänge um 7 Tage oder mehr in irgendeine Richtung (länger oder kürzer) gegenüber der ursprünglichen Zykluslänge für mindestens 2 Zyklen

1. Late transition: 3 to 11 months without menstruation

German: Späte Umstellung: 3 bis 11 Monate ohne Regelblutung (Menstruation)

1. Postmenopausal: 12 months or more without menstruation

German: Postmenopause: 12 Monate oder mehr ohne Regelblutung (Menstruation)

Have you had all or part of your uterus and/or ovaries removed, or are there other organic reasons for a lack of menstrual cycle? This may have an influence on the data collected in this study. [hysterectomy]

German: Sind bei Ihnen die Gebärmutter und/oder die Eierstöcke ganz oder teilweise entfernt worden oder liegen andere organische Gründe für einen fehlenden Menstruationszyklus vor? Dies kann Einfluss auf die in dieser Studie erhobenen Daten haben.

1. Nein (no)
2. Ja (yes)

How regular was your menstrual cycle in the last 12 months? [menstruation_regularity]

German: Wie regelmäßig war Ihr Menstruationszyklus in den letzten 12 Monaten?

1. regular, i.e. 28 days with a maximum of 3 days deviation (a cycle length of 25 to 31 days) for most cycle phases

German: Regelmäßig, das heißt 28 Tage mit maximal 3 Tagen Abweichung (eine Zykluslänge von 25 bis 31 Tagen) für die meisten Zyklusphasen

1. irregular, i.e. more than 3 days deviation (a cycle length of less than 25 or more than 31 days) for most cycle phases

German: Unregelmäßig, das heißt mehr als 3 Tagen Abweichung (eine Zykluslänge von weniger als 25 oder mehr als 31 Tagen) für die meisten Zyklusphasen

How many days have passed since the first day of your last menstrual period? If you use a cycle app, you can consult it. [last_menstruation]

German: Wie vielen Tage sind seit dem ersten Tag Ihrer letzten Monatsblutung vergangen? Wenn Sie eine Zyklus-App verwenden, können Sie diese zu Rate ziehen.

__ Tage (days)

The following measures were included to screen participants for eligibility and to perform additional analyses.

We included the German version of the to assess clinically relevant facets of dissociation using 44 items.

We included the German version of the *Dissociative Symptom Scale* (DSS; Carlson et al., 2018) to further assess clinically relevant facets of dissociation using 20 items. The scale was translated by Nikolaus Kleindienst (personal communication, April 04, 2023).

Instruction: For each statement below, check a box to show how much each thing has happened to you **in the last 2 weeks**. Please leave out of your answers episodes when you have been under the influence of alcohol, drugs or medication.

German: Bitte kreuzen Sie bei jeder der Aussagen an, wie häufig Sie dies **in den letzten 2 Woche** erlebt haben. Bitte lassen Sie bei Ihren Antworten Episoden unberücksichtigt, in denen Sie unter dem Einfluss von Alkohol, Drogen oder Medikamenten gestanden haben.

Scoring: 1 (never)

German: 0 (nie) 1 (einmal oder zweimal) 2 (fast täglich) 3 (etwa einmal täglich) 4 (mehr als einmal täglich)

1. My body feels strange or unreal. (DSS_1)

German: Mein Körper fühlte sich fremd oder unwirklich an.

1. Things around me seemed strange or unreal. (DSS_2) (DSS-B_1)

German: Die Dinge um mich herum wirkten fremd oder unwirklich.

1. I got reminded of something upsetting and then spaced out for a while. (DSS_3)

German: Ich wurde an etwas Aufwühlendes erinnert und war zeitweise wie weggetreten.

1. I had moments when I lost control and acted like I was back in an upsetting time in my past. (DSS_4) (DSS-B_2)

German: Es gab Momente, in denen ich die Kontrolle verlor und mich verhalten habe, als wäre ich wieder in einer belastenden Zeit in der Vergangenheit.

1. I felt like I was outside myself, watching myself doing things. (DSS_5)

German: Ich fühlte mich wie außerhalb meines Körpers und sah mir selbst von außen zu.

1. I heard something that I know really wasn't there. (DSS_6) (DSS-B_3)

German: Ich hörte etwas, von dem ich wusste, dass es gar nicht da war.

1. I got upset about something and can’t remember what happened next. (DSS_7)

German: Ich habe mich über etwas aufgeregt und kann mich nicht erinnern, was als nächstes geschah.

1. I felt like I was in a movie – like nothing that was happening was real. (DSS_8) (DSS-B_4)

German: Ich fühlte mich wie in einem Film – alles was passierte, wirkte unwirklich.

1. I didn’t feel pain when I was hurt and should have felt something. (DSS_9)

German: Ich spürte keine Schmerzen, obwohl ich mich verletzte und etwas hätte spüren müssen.

1. A memory came back to me that was so strong that I lost track of what was going on around me. (DSS_10)

German: Eine Erinnerung holte mich ein, die so stark war, dass ich nicht mehr mitbekam, was um mich herum vor sich ging.

1. I found myself staring into space and thinking of nothing. (DSS_11)

German: Ich fand mich wieder, wie ich ins Leere starrte, ohne irgendetwas zu denken.

1. I couldn’t remember things that had happened during the day even when I tried to. (DSS_12)

German: Ich konnte mich nicht an Dinge erinnern, die den Tag über passiert sind – selbst, wenn ich es versucht habe.

1. I felt like I wasn’t myself. (DSS_13)

German: Ich fühlte mich, als sei ich nicht ich selbst.

1. I saw something that seemed real, but was not. (DSS_14) (DSS-B_5)

German: Ich sah etwas, das mir wirklich erschien, obwohl es nicht da war.

1. I suddenly realized that I hadn’t been paying attention to what was going on around me. (DSS-B_6)

German: Mir fiel plötzlich auf, dass ich nicht bemerkt hatte, was um mich herum vorging.

1. Parts of my body seemed distorted – like they were bigger or smaller than usual. (DSS_16)

German: Teile meines Körpers schienen verzerrt – als ob sie größer oder kleiner wären als sonst.

1. I reacted to people or situations as if I were back in an upsetting time in my past. (DSS_17) (DSS-B_7)

German: Ich reagierte auf Menschen oder Situationen, als wäre ich wieder in einer belastenden Zeit in meiner Vergangenheit.

1. I got so focused on something going on in my mind that I lost track of what was happening around me. (DSS_18) (DSS-B_8)

German: Ich war so in meine Gedanken vertieft, dass ich nicht mitbekam, was um mich herum vor sich ging.

1. I noticed gaps in my memory of things that happened to me that I should be able to remember. (DSS_19)

German: Ich bemerkte Lücken in meiner Erinnerung bei Dingen, die mir passiert sind und an die ich mich eigentlich erinnern können müsste.

1. I smelled something that I knew wasn't there. (DSS_20)

German: Ich roch etwas, von dem ich wusste, dass es gar nicht da war.

We included the German version of the *Dissociative Experience Scale* (DES; Freyberger et al., 1998; Spitzer et al., 2021) to further assess clinically relevant facets of dissociation using 44 items.

Instruction: Please indicate the extent to which you have had the following experiences and episodes in the past 2 weeks. Please leave out of your answers episodes in your life when you have been under the influence of alcohol, drugs or medication.

German: Bitte geben Sie an, in welchem Ausmaß Sie die folgenden Erfahrungen und Erlebnisse in den letzten 2 Wochen hatten. Bitte lassen Sie bei Ihren Antworten Episoden in Ihrem Leben unberücksichtigt, in denen Sie unter dem Einfluss von Alkohol, Drogen oder Medikamenten gestanden haben.

Scoring: 0% (never) 10 20 30 40 50 60 70 80 90 100% (always)

German: 0% (niemals) 10 20 30 40 50 60 70 80 90 100% (immer)

1. People occasionally ride a car, bus, or subway and suddenly realize they can't remember what happened during the ride. [DES_1]

German: Menschen fahren gelegentlich mit einem Auto, einem Bus oder einer U-Bahn und stellen plötzlich fest, dass sie nicht daran erinnern können, was während der Fahrt geschehen ist.

1. People sometimes find that they are listening to someone and suddenly realize that they have heard nothing or only parts of what has been said. [DES_2]

German: Menschen stellen manchmal fest, dass sie jemanden zuhören und plötzlich erkennen, dass sie von dem, was gesagt worden ist, nichts oder nur Teile gehört haben.

1. It happens to people occasionally to find themselves in a place and not know they got there. [DES_3]

German: Menschen passiert es gelegentlich, sich an einem Ort zu befinden und nicht zu wissen, sie dorthin gekommen sind.

1. People sometimes find themselves wearing clothes without remembering putting them on. Please mark with your answer how often this happens to you. [DES_4]

German: Menschen stellen manchmal fest, Kleidungsstücke zu tragen, ohne sich daran zu erinnern, diese angezogen zu haben. Kennzeichnen Sie bitte mit Ihrer Antwort, wie häufig Ihnen dies passiert.

1. Sometimes people have difficulties seeing (e.g., double vision, veil sight, blindness on one or both eyes) without a doctor being able to find a physical cause. [DES_5] [derealization] (item only in the German version; Spitzer et al., 2021)

German: Manchmal haben Menschen Schwierigkeiten mit den Augen (z. B. Doppelt- oder Schleiersehen, blind auf einem oder beiden Augen), ohne dass ein Arzt eine körperliche Ursache finden konnte.

1. Sometimes people have the experience of feeling that their body does not seem to belong to them. [DES_6] [depersonalization] (also included in DES-Taxon)

German: Manchmal haben Menschen zeitweise das Gefühl, dass ihr Körper oder ein Teil ihres Körpers nicht zu ihnen gehört.

1. Sometimes people have the experience of feeling that other people, objects, and the world around them are not real. [DES_7] **[**derealization] (also included in DES-Taxon)

German: Manchmal haben Menschen zeitweise das Gefühl, als betrachteten sie die Welt durch einen Schleier, so dass Personen und Gegenstände wie weit entfernt, undeutlich oder unwirklich erscheinen.

1. Sometimes people occasionally feel like a robot. [DES_8] [depersonalization] (item only in the German version; Spitzer et al., 2021)

German: Manchmal fühlen Menschen sich gelegentlich wie ein Roboter.

1. Sometimes people believe they are being controlled by a spirit or magical force and they then behave completely differently than they normally would. [DES_9]

German: Manchmal glauben Menschen, von einem Geist oder einer magischen Kraft beherrscht zu werden und sie verhalten sich dann vollkommen anders als normalerweise.

1. People occasionally experience looking in the mirror and not recognizing themselves. [DES_10]

German: Menschen erleben gelegentlich, dass sie in den Spiegel schauen und sich nicht erkennen.

1. Sometimes people have the experience of feeling as though they are standing next to themselves or watching themselves do something, and they actually see themselves as though they were looking at another person. [DES_11] [depersonalization_ trait_2]

German: Manchmal machen Menschen die Erfahrung, neben sich zu stehen oder sich selbst zu beobachten, wie sie etwas tun; und dabei sehen sie sich selbst tatsächlich so, als ob sie eine andere Person betrachteten.

1. People sometimes believe that inside them there is another person for whom they have a name. [DES_12]

German: Menschen glauben manchmal, dass in ihrem Inneren eine andere Person existiert, für die sie einen Namen haben.

1. People occasionally have problems with hearing (e.g., annoying ringing in the ears, deafness in one or both ears) without a doctor being able to find a physical cause. [DES_13]

German: Menschen haben gelegentlich Probleme mit dem Hören (z. B. lästige Ohrgeräusche, taub auf einem oder beiden Ohren), ohne dass ein Arzt eine körperliche Ursache finden konnte.

1. People sometimes find themselves suddenly hoarse or unable to speak at all, without a doctor being able to find a physical cause. [DES_14]

German: Menschen passiert es zuweilen, dass sie plötzlich heiser sind oder gar nicht mehr sprechen können, ohne dass ein Arzt eine körperliche Ursache finden konnte.

1. People occasionally find that their handwriting (suddenly) changes completely. [DES_15]

German: Menschen stellen gelegentlich fest, dass sich (plötzlich) ihre Handschrift vollkommen verändert.

1. People are occasionally not sure whether events they remember really happened or whether they merely dreamed them. [DES_16]

German: Menschen sind sich gelegentlich nicht sicher, ob Ereignisse, an die sie sich erinnern, wirklich geschehen sind oder ob sie diese lediglich geträumt haben.

1. People sometimes notice that when they are watching TV or a movie, they get so caught up in the story that they don't notice other things happening around them. [DES_17]

German: Menschen bemerken zuweilen, dass sie beim Fernsehen oder Anschauen eines Films so in der Geschichte aufgehen, dass sie andere Dinge, die um sie herum geschehen, nicht wahrnehmen.

1. People sometimes find that they have done things that they can't remember. [DES_18]

German: Menschen stellen manchmal fest, dass sie Dinge getan haben, an die sie sich nicht erinnern können.

1. People sometimes find writing, drawings, or notes among their personal belongings that originated with them but that they do not remember making. [DES_19]

German: Menschen finden manchmal Schriftstücke, Zeichnungen oder Notizen unter ihren persönlichen Gegenständen, die von ihnen stammen, an deren Anfertigung sie sich jedoch nicht erinnern können.

1. People at times find that they have become so immersed in a fantasy story or daydream that they have the impression that these are really happening. [DES_20]

German: Menschen stellen zeitweise fest, dass sie sich so sehr in eine Phantasiegeschichte oder einen Tagtraum hineinversetzt haben, dass sie den Eindruck haben, diese geschähen wirklich.

1. People sometimes find that they hear certain voices in their head instructing them to do things or commenting on their actions. [DES_21]

German: Menschen stellen manchmal fest, dass sie bestimmte Stimmen in ihrem Kopf hören, die sie anweisen, Dinge zu tun, oder ihr Handeln kommentieren.

1. People sometimes find that they cannot remember important events in their lives, such as a wedding or a graduation ceremony. [DES_22]

German: Menschen stellen zuweilen fest, dass sie sich nicht an wichtige Ereignisse in ihrem Leben erinnern können, wie z. B. an eine Hochzeit oder an eine Schulabschlussfeier.

1. People sometimes no longer feel body parts or experience strange sensations such as burning, tingling or numbness without a doctor being able to find a physical cause. [DES_23]

German: Menschen spüren manchmal Körperteile nicht mehr oder erleben eigenartige Gefühle wie z. B. Brennen, Kribbeln oder Taubheit, ohne dass ein Arzt eine körperliche Ursache finden konnte.

1. It occasionally happens to people that they leave their familiar surroundings for hours or days without knowing the reason or occasion for doing so (without being able to remember for what reason or occasion they did so). [DES_24]

German: Menschen passiert es gelegentlich, dass sie für Stunden oder Tage ihre gewohnte Umgebung verlassen, ohne den Grund oder Anlass dafür zu wissen (ohne sich daran erinnern zu können, aus welchem Grund oder Anlass sie dies getan haben).

1. People occasionally find that their legs or arms are very weak or they can't move their limbs at all without a doctor being able to find a physical cause. [DES_25]

German: Menschen stellen gelegentlich fest, dass Ihre Beine oder Arme sehr schwach sind oder sie ihre Gliedmaßen gar nicht mehr bewegen können, ohne dass ein Arzt eine körperliche Ursache finden konnte.

1. It occasionally happens to people that they are accused of lying, although they themselves are firmly convinced that they have not lied. [DES_26]

German: Menschen passiert es gelegentlich, dass man ihnen vorwirft zu lügen, obwohl sie selbst der festen Überzeugung sind, nicht gelogen zu haben.

1. People are sometimes told that they do not recognize friends or family members. [DES_27]

German: Menschen wird manchmal gesagt, dass sie Freunde oder Familienangehörige nicht erkennen.

1. People sometimes notice that are able to ignore pain. [DES_28]

German: Menschen bemerken manchmal, dass die fähig sind, Schmerzen zu ignorieren.

1. It occasionally happens to people that they can no longer coordinate and control their movements (e.g., they miss) without a doctor being able to find a physical cause. [DES_29]

German: Menschen passiert es gelegentlich, dass sie ihre Bewegungen nicht mehr koordinieren und kontrollieren können (z. B. greifen sie daneben), ohne dass ein Arzt eine körperliche Ursache finden konnte.

1. People occasionally happen to find new things in their possession that they don't remember buying. [DES_30]

German: Menschen passiert es gelegentlich, neue Dinge in ihrem Besitz zu finden, an deren Kauf sie sich nicht erinnern können.

1. People sometimes find that they are approached by someone who calls them by a different name or who insists that they have met them before. [DES_31]

German: Menschen stellen manchmal fest, dass sich ihnen irgendjemand nähert, der sie mit einem anderen Namen anspricht oder der darauf besteht, sie bereits einmal getroffen zu haben.

1. People sometimes feel that other people, objects, and the world around them are not real. [DES_32]

German: Menschen haben zuweilen das Gefühl, dass andere Personen, Gegenstände und die Welt um sich herum nicht wirklich sind.

1. People sometimes experience talking out loud to themselves when they are alone. [DES_33]

German: Menschen erleben manchmal, dass sie, laut mit sich selbst sprechen, wenn sie alleine sind.

1. People occasionally experience that they cannot remember whether they really did something or merely thought about doing it (e.g., they do not know whether they really posted a letter or merely thought about posting it). [DES_34]

German: Menschen erleben gelegentlich, dass sie sich nicht erinnern können, ob sie etwas wirklich getan haben oder lediglich darüber nachgedacht haben, es zu tun (z. B. wissen sie nicht, ob sie einen Brief wirklich eingeworfen haben oder lediglich darüber nachgedacht haben, ihn einzuwerfen).

1. It sometimes happens to people that they sit almost completely motionless for hours or days, almost do not speak, almost do not move and also do not react properly to external stimuli, such as loud noises. [DES_35]

German: Menschen passiert es zuweilen, dass sie stunden- oder tagelang fast völlig bewegungslos dasitzen, fast nicht sprechen, sich fast nicht bewegen und auch auf äußere Reize, wie z. B. laute Geräusche, nicht richtig reagieren.

1. People occasionally pass out without a doctor being able to find a physical cause. [DES_36]

German: Menschen werden gelegentlich bewusstlos, ohne dass ein Arzt eine körperliche Ursache finden konnte.

1. People sometimes find that in certain situations they are able to do things with amazing ease and spontaneity that they would normally find difficult (e.g., sports, work, social situations). [DES_37]

German: Menschen stellen manchmal fest, dass sie in bestimmten Situationen in der Lage sind, Dinge mit erstaunlicher Leichtigkeit und Spontaneität zu tun, die ihnen normalerweise schwer fallen würden (z. B. Sport, Arbeit, soziale Situationen).

1. People sometimes remember a past event so vividly that they feel they are reliving it. [DES_38]

German: Menschen erinnern sich manchmal so lebhaft an ein vergangenes Ereignis, dass sie das Gefühl haben, dieses Ereignis erneut zu erleben.

1. People sometimes find that they just sit and stare into space, thinking of nothing and not noticing how time passes. [DES_39]

German: Menschen stellen manchmal fest, dass sie einfach dasitzen und ins Leere starren, an nichts denken und nicht bemerken, wie die Zeit vergeht.

1. People occasionally experience becoming unsteady when standing or walking, making strange movements, or suddenly being unable to move at all without a doctor being able to find a physical cause. [DES_40]

German: Menschen erleben gelegentlich, wie sie beim Stehen oder Gehen unsicher werden, eigenartige Bewegungen machen oder sich plötzlich gar nicht mehr bewegen können, ohne dass ein Arzt eine körperliche Ursache finden konnte.

1. People sometimes find themselves in a familiar place and yet experience it as foreign and unfamiliar. [DES_41]

German: Menschen stellen manchmal fest, an einem vertrauten Ort zu sein und ihn dennoch als fremd und unbekannt zu erleben.

1. People occasionally find that they act so differently in comparable situations that they feel they are two different people. [DES_42]

German: Menschen stellen gelegentlich fest, dass sie in vergleichbaren Situationen so unterschiedlich handeln, dass sie das Gefühl haben, zwei unterschiedliche Personen zu sein.

1. People sometimes experience seizures without a doctor being able to find a physical cause. [DES_43]

German: Menschen erleiden manchmal Krampfanfälle, ohne dass ein Arzt eine körperliche Ursache finden konnte.

1. Sometimes people temporarily feel that body parts change (in size) (e.g., their arms become misshapen or larger and larger). [DES_44]

German: Manchmal haben Menschen zeitweise das Gefühl, dass sich Körperteile (in ihrer Größe) verändern (z. B. werden ihre Arme unförmig oder immer größer).

The following measures were included to perform additional analyses.

We included the German version of the *Difficulties in Emotion Regulation Scale – Short Form* (DERS-SF; Gutzweiler & In-Albon, 2019; Kaufman et al., 2015) to assess deficits in emotion regulation using 18 items.

Instruction: This questionnaire asks about how you respond to your emotions. Please score the following statements according to how much you agree or disagree that the statement is true of you. The questions mention *upsetting* feelings, this means emotions like sadness, anger, or fear.

German: In diesem Fragebogen wird erfasst, wie Sie auf Ihre Emotionen reagieren. Bitte geben Sie für die folgenden Aussagen an, wie sehr Sie zustimmen oder nicht zustimmen, dass diese auf Sie zutreffen. In den Fragen geht es um *negative* Gefühle wie Traurigkeit, Wut oder Angst.

Scoring: 1 (almost never) – 5 (almost always)

German: 1 (sehr selten) – 5 (sehr häufig)

1. I have difficulty making sense out of my feelings. [clarity_1]

German: Ich habe Schwierigkeiten, meine Gefühle zu verstehen.

1. I have no idea how I am feeling. [clarity_2]

German: Ich weiß nicht, wie ich mich fühle.

1. I am confused about how I feel. [clarity_3]

German: Ich bin über meine Gefühle verwirrt.

1. I care about what I am feeling. [awareness_1]

German: Ich achte auf meine Gefühle.

1. I pay attention to how I feel. [awareness_3]

German: Ich achte darauf, wie ich mich fühle.

When I’m upset, …

German: Wenn ich negative Gefühle habe, …

1. … I acknowledge my emotions. [awareness_2]

German: … erkenne ich meine Gefühle an.

1. … I become out of control. [control_1]

German: … gerate ich außer Kontrolle.

1. … I have difficulty controlling my behaviors. [control_2]

German: … habe ich Schwierigkeiten, mein Verhalten zu kontrollieren.

1. … I lose control over my behaviors. [control_3]

German: … verliere ich die Kontrolle über mein Verhalten.

1. … I become embarrassed for feeling that way. [non-acceptance_1]

German: … ist es mir peinlich, dass ich mich so fühle.

1. … I feel guilty for feeling that way. [non-acceptance_2]

German: … habe ich Schuldgefühle, so zu empfinden.

1. … I become irritated at myself for feeling that way. [non-acceptance_3]

German: … ärgere ich mich über mich selbst, dass ich so fühle.

1. … I have difficulty focusing on other things. [goals_1]

German: … habe ich Schwierigkeiten, über etwas anderes nachzudenken.

1. … I have difficulty concentrating. [goals_2]

German: … habe ich Schwierigkeiten, mich zu konzentrieren.

1. …I have difficulty getting work done. [goals_3]

German: … habe ich Schwierigkeiten, meine Arbeit zu schaffen.

1. … it takes me a long time to feel better. [strategies_1]

German: … brauche ich lange, um mich wieder besser zu fühlen.

1. … I believe that there is nothing I can do to make myself feel better. [strategies_2]

German: … glaube ich, dass ich nichts tun kann, um mich besser zu fühlen.

1. … I believe that I’ll end up feeling very depressed. [strategies_3]

German: … glaube ich, dass ich am Ende sehr niedergeschlagen sein werde.

We included the German version of the *Process Model of Emotion Regulation Questionnaire* (PMERQ; Olderbak et al., 2022) to assess individual differences in emotion regulation using 45 items.

Instructions: We are interested in which strategies people use to regulate their emotions, specifically to decrease the negative emotion that they feel. Please rate your agreement with the following statements using the response options listed below. There is no right or wrong answer.

German: Uns interessiert, welche Strategien Sie anwenden, um Ihre Emotionen zu regulieren, insbesondere um die negativen Emotionen, die Sie empfinden, zu verringern. Bitte bewerten Sie Ihre Zustimmung zu den folgenden Aussagen anhand der unten aufgeführten Antwortmöglichkeiten. Es gibt keine richtige oder falsche Antwort.

Scoring: 1 (strongly disagree) 2 (disagree) 3 (somewhat disagree) 4 (somewhat agree) 5 (agree) 6 (strongly agree)

German: 1 (Stimme überhaupt nicht zu) 2 (Stimme nicht zu) 3 (Stimme eher nicht zu) 4 (Stimme eher zu) 5 (Stimme zu) 6 (Stimme völlig zu)

1. To calm down, I do not show others how I feel. [expressive_ suppression_1]

German: Um mich zu beruhigen, zeige ich anderen nicht, wie ich mich fühle.

1. I concentrate on the least negative aspects of an upsetting situation, to feel less upset. [focus_elsewhere_1]

German: Ich konzentriere mich auf die am wenigsten negativen Aspekte einer aufwühlenden Situation, um mich weniger aufzuregen.

1. To feel less anxious during a conflict, I move the conversation to another topic. [avoid_conflict_1]

German: Um mich während eines Konflikts weniger ängstlich zu fühlen, verlagere ich das Gespräch auf ein anderes Thema.

1. To feel less anxious, I avoid stressful situations. [avoid_upsituations_1]

German: Um mich weniger ängstlich zu fühlen, vermeide ich stressige Situationen.

1. I avoid situations others tell me will be unpleasant, to feel less bad. [avoid_upsituations_2]

German: Ich vermeide Situationen, von denen mir andere sagen, dass sie unangenehm sein werden, um mich weniger schlecht zu fühlen.

1. I work to negotiate a resolution to conflicts I have with others, to decrease how bad I feel. [resolve_conflict_1]

German: Ich arbeite daran eine Lösung für Konflikte, die ich mit anderen habe, auszuhandeln, um zu verringern, wie schlecht ich mich fühle.

1. To feel less upset when things do not go as planned, I think about what may be the benefits. [consider_benefits_1]

German: Um mich weniger aufzuregen, wenn die Dinge nicht so laufen wie geplant, denke ich darüber nach, was die Vorteile sein können.

1. I express how I feel to my friends as a way to feel less bad. [emotional_sharing_1]

German: Ich drücke meinen Freundinnen/Freunden gegenüber aus, wie ich mich fühle, als ein Weg, um mich weniger schlecht zu fühlen.

1. When I have something unpleasant to discuss with someone, I confront them to feel less bad. [confront_upsituations_1]

German: Wenn ich mit jemandem etwas Unangenehmes zu diskutieren habe, stelle ich sie/ihn zur Rede, um mich weniger schlecht zu fühlen.

1. During conflicts I change the topic towards something less upsetting, to feel less bad. [avoid_conflict_2]

German: Während Konflikte wechsle ich das Thema zu etwas weniger Aufwühlendem, um mich weniger schlecht zu fühlen.

1. I confront upsetting situations head-on to feel less upset. [confront_upsituations_2]

German: Ich konfrontiere aufwühlende Situationen direkt, um mich weniger aufzuregen.

1. To feel less bad, I avoid people if I expect an interaction with them to be unpleasant. [avoid_upsituations_3]

German: Um mich weniger schlecht zu fühlen, gehe ich Menschen aus dem Weg, wenn ich erwarte, dass eine Interaktion mit ihnen unangenehm sein wird.

1. To feel less anxious in stressful situations, I divert my attention away from the situation. [cognitively_distract_1]

German: Um mich in stressigen Situationen weniger ängstlich zu fühlen, lenke ich meine Aufmerksamkeit von der Situation ab.

1. I steer contentious conversations towards a different topic, to reduce how upset they make me feel. [avoid_conflict_3]

German: Ich lenke strittige Gespräche auf ein anderes Thema, um zu reduzieren, wie sehr sie mich aufregen.

1. To feel less anxious, I tackle stressful situations head-on. [confront_upsituations_3]

German: Um mich weniger ängstlich zu fühlen, packe ich stressige Situationen direkt an.

1. I suppress my emotion expressions during stressful conversations to feel less anxious. [expressive_ suppression_2]

German: Ich unterdrücke meine Gefühlsausdrücke während stressiger Gespräche, um mich weniger ängstlich zu fühlen.

1. To reduce how upset conflicts make me, I actively find compromises. [resolve_conflict_2]

German: Um zu verringern, wie sehr mich Konflikte aufregen, finde ich aktiv Kompromisse.

1. I avoid stressful situations, to prevent feeling anxious. [avoid_upsituations_4]

German: Ich vermeide stressige Situationen, um zu verhindern, dass ich mich ängstlich fühle.

1. During a conflict, to calm myself down I work towards finding a compromise. [resolve_conflict_3]

German: Um mich während eines Konflikts zu beruhigen, arbeite ich daran, einen Kompromiss zu finden.

1. To reduce how upset I feel when something upsetting happens, I think of this as a chance to grow. [consider_benefits_2]

German: Um zu verringern, wie sehr ich mich aufrege, wenn etwas Aufwühlendes passiert, denke ich, dass es eine Chance ist, zu wachsen.

1. To feel less nervous during a stressful situation, I think about the good things that could come from the situation. [consider_benefits_3]

German: Um mich während einer stressigen Situation weniger nervös zu fühlen, denke ich über die guten Dinge nach, die sich durch die Situation ergeben könnten.

1. To reduce how bad I feel during unpleasant conversations, I focus on anything the person says that is not unpleasant. [focus_elsewhere_2]

German: Um zu verringern, wie schlecht ich mich während unangenehmer Gespräche fühle, fokussiere ich mich auf alles, was die Person sagt, das nicht unangenehm ist.

1. I focus on the least negative components of a stressful situation, to feel less anxious. [focus_elsewhere_3]

German: Ich fokussiere mich auf die am wenigsten negativen Komponenten einer stressigen Situation, um mich weniger ängstlich zu fühlen.

1. To feel less upset when things do not work out as planned, I think of it as a chance to learn. [consider_benefits_4]

German: Um mich weniger aufzuregen, wenn die Dinge nicht so funktionieren wie geplant, denke ich, es ist eine Chance zu lernen.

1. When something does not go as planned, I re-evaluate its importance to reduce how bad I feel. [reduce_importance_2]

German: Wenn etwas nicht so läuft wie geplant, bewerte ich dessen Wichtigkeit neu, um zu verringern, wie schlecht ich mich fühle.

1. During conflicts, I calm down by negotiating a resolution to the conflict. [resolve_conflict_4]

German: Während Konflikte beruhige ich mich, indem ich eine Lösung für den Konflikt aushandle.

1. I distract myself during unpleasant situations to feel less bad. [cognitively_distract_2]

German: Ich lenke mich während unangenehmer Situationen ab, um mich weniger schlecht zu fühlen.

1. To reduce how upset a situation makes me feel, I tackle it head-on. [confront_upsituations_4]

German: Um zu verringern, wie sehr mich eine Situation aufregt, packe ich sie direkt an.

1. To feel less upset during upsetting situations, I divert my attention away from what is happening. [cognitively_distract_3]

German: Um mich in aufwühlenden Situationen weniger aufzuregen, lenke ich meine Aufmerksamkeit von dem, was passiert, ab.

1. To feel less stressed, I ask others for help. [emotional_sharing_2]

German: Um mich weniger gestresst zu fühlen, bitte ich andere um Hilfe.

1. To reduce how anxious I feel during stressful conversations, I focus on things the person says that are not negative. [focus_elsewhere_4]

German: Um zu verringern, wie ängstlich ich mich während stressiger Gespräche fühle, fokussierte ich mich auf Dinge, die die Person sagt, die nicht negativ sind.

1. I talk with others about what makes me nervous, to feel less anxious. [emotional_sharing_3]

German: Ich spreche mit anderen darüber, was mich nervös macht, um mich weniger ängstlich zu fühlen.

1. To reduce how bad I feel during unpleasant situations, I do something to distract myself. [cognitively_distract_4]

German: Um zu verringern, wie schlecht ich mich während unangenehmer Situationen fühle, tue ich etwas, um mich abzulenken.

1. I do not tell others when I am upset, as a way to reduce how upset I feel. [expressive_ suppression_3]

German: Ich sage anderen nicht, wenn ich mich aufrege, als ein Weg um zu verringern, wie sehr ich mich aufrege.

1. During stressful conversations, I distract myself to feel less anxious. [cognitively_distract_5]

German: Während stressiger Gespräche lenke ich mich ab, um mich weniger ängstlich zu fühlen.

1. To reduce how bad conflicts make me feel, I work to solve the disagreement. [resolve_conflict_5]

German: Um zu verringern, wie schlecht mich Konflikte fühlen lassen, arbeite ich daran, die Meinungsverschiedenheit zu lösen.

1. To feel less upset during a heated conversation, I change the subject. [avoid_conflict_4]

German: Um mich während eines hitzigen Gesprächs weniger aufzuregen, wechsle ich das Thema.

1. If something does not work out as I wanted, to feel less bad I decide that perhaps it was not so important. [reduce_importance_3]

German: Falls etwas nicht so klappt, wie ich es wollte, beschließe ich, um mich weniger schlecht zu fühlen, dass es vielleicht nicht so wichtig war.

1. I work to find a solution to conflicts, to decrease how anxious they make me feel. [resolve_conflict_6]

German: Ich arbeite daran, eine Lösung für Konflikte zu finden, um zu verringern, wie ängstlich sie mich fühlen lassen.

1. When something does not work out, to calm down I try to see it as a blessing in disguise. [consider_benefits_5]

German: Wenn etwas nicht klappt, versuche ich, um mich zu beruhigen, es als Glück im Unglück zu sehen.

1. When going for something I want gets me in a stressful situation, to feel less anxious I question the importance of what I want. [reduce_importance_4]

German: Wenn ich in eine stressige Situation gerate, weil ich etwas erreichen möchte, hinterfrage ich, um mich weniger ängstlich zu fühlen, die Wichtigkeit dessen, was ich möchte.

1. To reduce how upset a situation makes me feel, I avoid the situation. [avoid_upsituations_5]

German: Um zu verringern, wie sehr mich eine Situation aufregt, vermeide ich die Situation.

1. I steer combative conversations to neutral topics, to reduce how bad they make me feel. [avoid_conflict_5]

German: Ich lenke hitzige Gespräche auf neutrale Themen, um zu verringern, wie schlecht sie mich fühlen lassen.

1. I avoid upsetting conversations to feel less upset. [avoid_upsituations_6]

German: Ich vermeide aufwühlende Gespräche, um mich weniger aufzuregen.

1. When something upsetting happens, to feel less upset, I think about the possible benefits of the situation. [consider_benefits_6]

German: Wenn etwas Aufwühlendes passiert, denke ich, um mich weniger aufzuregen, über die möglichen Vorteile der Situation nach.

We included the German version of the *Childhood Trauma Questionnaire* (CTQ; Wingenfeld et al., 2010) to retrospectively assess self-reported childhood trauma using 25 items.

Instructions: The following questions relate to your childhood and youth. Even though the questions are very personal, we would like you to answer as honestly as possible. There are no right or wrong answers. Please tick the number behind each question that best applies to you.

German: Die folgenden Fragen beziehen sich auf Ihre Kindheit und Jugend. Auch wenn die Fragen sehr persönlich sind, möchten wir Sie bitten, so ehrlich wie möglich zu antworten. Es gibt keine richtigen oder falschen Antworten. Bitte kreuzen Sie hinter jeder Frage die Zahl an, die auf Sie am besten zutrifft.

Scoring: 1 (never) – 5 (very often)

German: 1 (niemals) – 5 (sehr häufig)

When I was growing up ...

German: Als ich aufwuchs ...

1. … there was someone in my family who helped me feel important or special. [emotional_neglect_1r]

German: … gab es jemand in der Familie, der mir das Gefühl gab, wichtig und jemand Besonderes zu sein.

1. … I felt loved. [emotional_neglect_2r]

German: … hatte ich das Gefühl, geliebt zu werden.

1. … people in my family looked out for each other. [emotional_neglect_3r]

German: ... gaben meine Familienangehörigen aufeinander acht.

1. … people in my family felt close to each other. [emotional_neglect_4r]

German: ... fühlten sich meine Familienangehörigen einander nah.

1. … my family was a source of strength and support. [emotional_neglect_5r]

German: ... war meine Familie mir eine Quelle der Unterstützung.

1. … I didn't have enough to eat. [physical_neglect_1]

German: ... hatte ich nicht genug zu essen.

1. … [physical_neglect_2r]

German: … wusste ich, dass sich jemand um mich sorgte und mich beschützte.

1. … my parents were too drunk or high to take care of the family. [physical_neglect_3]

German: ... … waren meine Eltern zu betrunken oder von anderen Drogen „high“, um für die Familie zu sorgen.

1. … I had to wear dirty clothes. [physical_neglect_4]

German: ... musste ich dreckige Kleidung tragen.

1. … there was somebody to take me to the doctor if I needed it. [physical_neglect_5r]

German: … gab es jemanden, der mich zum Arzt brachte, wenn ich es brauchte.

1. … people in my family called me things like "stupid", "lazy", or "ugly". [emotional_abuse_1]

German: … bezeichneten mich Personen aus meiner Familie als „dumm“, „faul “ oder „hässlich“.

1. … I thought that my parents wished I had never been born. [emotional_abuse_2]

German: … glaubte ich, dass meine Eltern wünschten, ich wäre nie geboren.

1. ... people in my family said hurtful or insulting things to me. [emotional_ abuse_3]

German: … sagten Personen aus meiner Familie verletzende oder beleidigende Dinge zu mir.

1. … someone in my family hated me. [emotional_ abuse_4]

German: … hatte ich das Gefühl, es hasste mich jemand in meiner Familie.

1. … I got hit so hard by someone in my family that I had to see a doctor or go to the hospital. [physical_ abuse_1]

German: … wurde ich von jemandem aus meiner Familie so stark geschlagen, dass ich zum Arzt oder ins Krankenhaus musste.

1. ... people in my family hit me so hard that it left me with bruises or marks. [physical_ abuse_2]

German: … schlugen mich Personen aus meiner Familie so stark, dass ich blaue Flecken oder Schrammen davontrug.

1. … I was punished with a belt, a board, a cord (or some other hard object). [physical_ abuse_3]

German: … wurde ich mit einem Gürtel, einem Stock, einem Riemen oder mit einem harten Gegenstand bestraft.

1. … I got hit or beaten so badly that it was noticed by someone like a teacher, neighbor, or doctor. [physical_ abuse_4]

German: … wurde ich so stark geschlagen oder verprügelt, dass es jemandem (z. B. Lehrer, Nachbar oder Arzt) auffiel.

1. … someone tried to touch me in a sexual way or tried to make me touch them. [sexual_ abuse_1]

German: … versuchte jemand, mich sexuell zu berühren oder mich dazu zu bringen, sie oder ihn sexuell zu berühren.

1. … someone threatened to hurt me or tell lies about me unless I did something sexual with them. [sexual_ abuse_2]

German: … drohte mir jemand, mir weh zu tun oder Lügen über mich zu erzählen, wenn ich keine sexuellen Handlungen mit ihm oder ihr ausführen würde.

1. … someone tried to make me do sexual things or watch sexual things. [sexual_ abuse_3]

German: … versuchte jemand, mich dazu zu bringen, sexuelle Dinge zu tun oder bei sexuellen Dingen zuzusehen.

1. … someone molested me (took advantage of me sexually). [sexual_ abuse_4]

German: … belästigte mich jemand sexuell.

Scoring: 1 (strongly disagree) – 5 (strongly agree)

German: 1 (Stimme überhaupt nicht zu) – 5 (Stimme völlig zu)

I think…

Ich glaube…

1. … [emotional_abuse_5]

German: … ich bin emotional (gefühlsmäßig) missbraucht worden, als ich aufwuchs.

1. … [physical_abuse_5]

German: … ich bin körperlich misshandelt worden, als ich aufwuchs.

1. … [sexual_abuse_5]

German: … ich bin sexuell missbraucht worden, als ich aufwuchs.

We included a subscale of the German version of the *Posttraumatic Diagnostic Scale for DSM-5* (PDS-5; Wittmann et al., 2021) to assess self-reported reexperiencing and avoidance of any trauma-related memories using 7 items.

Instruction: Below is a list of problems that people sometimes experience after a traumatic event. Please read each of the statements below carefully and check off the number that best describes how often the problem occurred and how much stress it caused them in the last month. Rate each problem in relation to any traumatic event.

German: Nachfolgend finden Sie eine Liste an Problemen, die Menschen nach einem traumatischen Ereignis manchmal haben. Bitte lesen Sie jede der nachfolgenden Aussagen sorgfältig durch und kreuzen Sie die Zahl an, die am besten beschreibt, wie oft das Problem aufgetreten ist und wie stark sie dadurch im letzten Monat belastet waren. Schätzen Sie jedes Problem in Bezug auf *irgendein* traumatische Ereignis ein.

Scoring: (0) not at all (1) once a week or less often (2) 2-3x per week (3) 4-5x per week (4) more than 5x per week

German: (0) gar nicht (1) einmal pro Woche oder seltener (2) 2-3x pro Woche (3) 4-5x pro Woche (4) mehr als 5x pro Woche

1. Having upsetting thoughts or images about *any* traumatic event that came into your head when you didn’t want them to.

German: Ungewollte, belastende Erinnerungen an *irgendein* Trauma

1. Having bad dreams or nightmares about *any* traumatic event.

German: Schlechte Träume oder Albträume in Bezug auf *irgendein* Trauma

1. Reliving *any* traumatic event, acting or feeling as if it was happening again.

German: Wiedererleben *irgendeines* traumatischen Ereignisses oder Fühlen, als würde es tatsächlich wieder passieren

1. Feeling emotionally upset when you were reminded of *any* traumatic event (e.g., feeling scared, sad, angry, guilty etc.).

German: Sich EMOTIONAL sehr belastet fühlen, wenn Sie an *irgendein* Trauma erinnert werden

1. Experiencing physical reactions when you were reminded of the traumatic event (e.g., breaking out in a sweat, heart beating fast).

German: KÖRPERLICHE Reaktionen (z. B. Schwitzen, Herzrasen), wenn Sie an *irgendein* Trauma erinnert werden

1. Trying not to think about, or have feelings about *any* traumatic event.

German: Versuchen, Gedanken oder Gefühle in Bezug auf *irgendein* Trauma zu vermeiden

1. Trying to avoid activities, people or places that remind you of *any* traumatic event.

German: Versuchen, Aktivitäten, Situationen oder Orte zu vermeiden, die Sie an *irgendein* Trauma erinnern oder die seit *irgendeinem* Trauma gefährlicher erscheinen

The following measures were included to describe the psychopathological status our sample.

We included the German version of the *Patient Health Questionnaire-8* (PHQ-8; Kroenke et al., 2008; Löwe, 2015), which consists of eight items assessing the severity of depressive symptoms. The PHQ-8 is shorter than the Beck’s Depression Inventory but still demonstrates appropriate psychometric properties.

Instruction: Over the last 2 weeks, how often have you been bothered by any of the following problems?

German: Wie oft fühlten Sie sich im Verlauf der letzten 2 Wochen durch die folgenden Beschwerden beeinträchtigt?

Scoring: (0) not at all – (1) several days – (2) more than half the days – (3) nearly every day

German: (0) überhaupt nicht – (1) an einzelnen Tagen – (2) an mehr als der Hälfte der Tage – (3) beinahe jeden Tag

1. Little interest or pleasure in doing things.

German: Wenig Interesse oder Freude an Ihren Tätigkeiten.

1. Feeling down, depressed, or hopeless.

German: Niedergeschlagenheit, Schwermut oder Hoffnungslosigkeit.

1. Trouble falling or staying asleep, or sleeping too much.

German: Schwierigkeiten ein- oder durchzuschlafen oder vermehrter Schlaf.

1. Feeling tired or having little energy.

German: Müdigkeit oder Gefühl, keine Energie zu haben.

1. Poor appetite or overeating.

German: Verminderter Appetit oder übermäßiges Bedürfnis zu essen.

1. Feeling bad about yourself - or that you are a failure or have let yourself or your family down.

German: Schlechte Meinung von sich selbst; Gefühl, ein Versager zu sein oder die Familie enttäuscht zu haben.

1. Trouble concentrating on things, such as reading the newspaper or watching television.

German: Schwierigkeiten, sich auf etwas zu konzentrieren, z.B. beim Zeitunglesen oder Fernsehen.

1. Moving or speaking so slowly that other people could have noticed? Or the opposite - being so fidgety or restless that you have been moving around a lot more than usual.

German: Waren Ihre Bewegungen oder Ihre Sprache so verlangsamt, dass es auch anderen auffallen würde? Oder waren Sie im Gegenteil „zappelig“ oder ruhelos und hatten dadurch einen stärkeren Bewegungsdrang als sonst?

We included the German version of the *Personality Inventory for DSM-5, Brief Form Plus* (PID5BF+M; Kerber et al., 2022) to assess self-reported psychopathological personality trait facets using 36 items.

Instruction: This is a list of things different people might say about themselves. We are interested in how you would describe yourself. There are no “right” or “wrong” answers. So you can describe yourself as honestly as possible, we will keep your responses confidential. Select the response that best describes you.

German: Im Folgenden finden Sie eine Liste von Aussagen, mit denen sich Menschen selbst beschreiben können. Wir interessieren uns dafür, wie Sie sich selbst beschreiben würden. Es gibt keine richtigen oder falschen Antworten. Bitte beschreiben Sie sich so ehrlich wie möglich – wir werden Ihre Antworten vertraulich behandeln. Kreuzen Sie jeweils diejenige Antwort an, die Sie am besten beschreibt.

Scoring: (0) very false or often false (1) sometimes or somewhat false (2) sometimes or somewhat true (3) very true or often true

German: (0) trifft überhaupt nicht zu (1) trifft eher nicht zu (2) trifft eher zu (3) trifft genau zu

1. I have much stronger emotional reactions than almost everyone else. (NA_liability_1)

German: Ich reagiere viel emotionaler als fast alle anderen Menschen.

1. I’m good at conning people. (AN_manipulativeness_1)

German: Ich bin gut darin, Leute reinzulegen.

1. I’m often pretty careless with my own and others’ things. (DI_irresponsibility_1)

German: Ich gehe oft ziemlich nachlässig mit meinen Sachen und denen anderer um.

1. I keep my distance from people. (DT_withdrawal_1)

German: Ich halte Abstand zu Menschen.

1. I often see unusual connections between things that most people miss. (PY_beliefs_1)

German: Ich sehe zwischen den Dingen oft ungewöhnliche Zusammenhänge, die anderen Menschen entgehen.

1. Even though it drives other people crazy, I insist on absolute perfection in everything I do. (AK_perfectionism_1)

German: Auch wenn es andere zum Wahnsinn treibt, bestehe ich darauf, alles perfekt zu machen.

1. I’m always worrying about something. (NA_anxiousness_1)

German: Ich mache mir ständig über irgendetwas Sorgen.

1. Sometimes you need to exaggerate to get ahead. (AN_deceitfulness_1)

German: Manchmal muss man vor anderen übertreiben, um weiterzukommen.

1. I feel like I act totally on impulse. (DI_impulsivity_1)

German: Es kommt mir vor, als würde ich völlig impulsiv handeln.

1. Nothing seems to interest me very much. (DT_anhedonia_1)

German: Nichts scheint mich wirklich zu interessieren.

1. People have told me that I think about things in a really strange way. (PY_eccentricity_1)

German: Man hat mir gesagt, dass meine Art zu denken wirklich seltsam ist.

1. It is important to me that things are done in a certain way. (AK_rigidity_1)

German: Es ist mir wichtig, dass Dinge auf eine ganz bestimmte Weise erledigt werden.

1. I worry a lot about being alone. (NA_separation_1)

German: Ich mache mir viele Sorgen darüber, allein zu sein.

1. I deserve special treatment. (AN_ grandiosity_1)

German: Es steht mir zu, besonders behandelt zu werden.

1. I lose track of conversations because other things catch my attention. (DI_distractibility_1)

German: Ich verliere in Gesprächen den Faden, weil mich andere Dinge ablenken.

1. I prefer to keep romance out of my life. (DT_intimacy_1)

German: Ich halte romantische Gefühle lieber aus meinem Leben heraus.

1. It’s weird, but sometimes ordinary objects seem to be in a different shape than usual. (PY_perceptual_1)

German: Es ist komisch, aber manchmal kommen mir alltägliche Gegenstände anders vor als sonst.

1. I keep trying to make things perfect, even when I’ve gotten them as good as they’re likely to get. (AK_perfectionism_2)

German: Ich versuche Dinge weiter zu perfektionieren, auch wenn ich sie wahrscheinlich schon so gut wie möglich hinbekommen habe.

1. I get emotional over every little thing. (NA_liability_2)

German: Ich werde schnell emotional, oft aus geringstem Anlass.

1. It is easy for me to take advantage of others. (AN_ manipulativeness_2)

German: Es fällt mir leicht, andere auszunutzen.

1. I often forget to pay my bills. (DI_irresponsibility_2)

German: Ich vergesse oft, meine Rechnungen zu bezahlen.

1. I don’t like spending time with others. (DT_withdrawal_2)

German: Ich mag es nicht, Zeit mit anderen zu verbringen.

1. I’ve had some really weird experiences that are very difficult to explain. (PY_beliefs_2)

German: Ich hatte einige wirklich seltsame Erlebnisse, die sehr schwer zu erklären sind.

1. I have a strict way of doing things. (AK_rigidity_2)

German: Ich habe für viele alltägliche Verhaltensweisen strikte Regeln.

1. I worry about almost everything. (NA_ anxiousness_2)

German: Ich mache mir über fast alles Sorgen.

1. I’ll stretch the truth if it’s to my advantage. (AN_deceitfulness_2)

German: Ich biege mir die Wahrheit zurecht, wenn es zu meinem Vorteil ist.

1. Even though I know better, I can’t stop making rash decisions. (DI_impulsivity_2)

Obwohl ich es eigentlich besser weiß, treffe ich immer wieder überstürzte Entscheidungen.

1. I rarely get enthusiastic about anything. (DT_anhedonia_2)

German: Ich bin selten von irgendetwas begeistert.

1. I have several habits that others find eccentric or strange. (PY_eccentricity_2)

German: Ich habe mehrere Angewohnheiten, die andere exzentrisch oder seltsam finden.

1. I’ve been told that I spend too much time making sure things are exactly in place. (AK_order_1)

German: Mir wurde schon mal gesagt, dass ich zu viel Zeit damit verbringe, darauf zu

achten, dass alles genau an seinem Platz ist.

1. I can’t stand being left alone, even for a few hours. (NA_separation_2)

German: Ich ertrage es nicht, allein gelassen zu werden – nicht mal für ein paar Stunden.

1. I often have to dealt with people who are less important than me. (AN_ grandiosity_2)

German: Ich muss mich oft mit Leuten beschäftigen, die weniger wichtig sind als ich.

1. I am easily distracted. (DI_distractibility_2)

German: Ich lasse mich leicht ablenken.

1. I break off relationships if they start to get close. (DT_intimacy_2)

German: Ich beende Beziehungen, wenn sie enger werden.

1. Sometimes when I look at a familiar object, it’s somehow like I’m seeing it for the first time. (PY_perceptual_2)

German: Wenn ich einen vertrauten Gegenstand anschaue, ist es manchmal so, als würde ich ihn zum ersten Mal sehen.

1. People complain about my need to have everything all arranged. (AK_order_2)

German: Andere beschweren sich darüber, dass bei mir alles genau durchgeplant sein muss.

Personality trait domains: NA = negative affect, DT = detachment, AN = antagonism, DI = disinhibition, AK = anankasm, PY = psychoticism.

The following measures were included to predict missingness in the experience sampling data sets.

We included the German 10 Item Big Five Inventory (BFI-10; Rammstedt et al., 2013).

Instruction: To what extent do the following statements apply to you?

German: Inwieweit treffen die folgenden Aussagen auf Sie zu?

Scoring: (1) does not apply at all (2) rather does not apply (3) neither/nor (4) rather applies (5) completely applies

German: (1) trifft überhaupt nicht zu (2) trifft eher nicht zu (3) weder/noch (4) eher zutreffend (5) trifft voll und ganz zu

1. I am rather withdrawn, reserved. (extraversion_1_r)

German: Ich bin eher zurückhaltend, reserviert.

1. I trust others easily, I believe in the goodness in people. (agreeableness_1)

German: Ich schenke anderen leicht Vertrauen, glaube an das Gute

im Menschen.

1. I am comfortable, prone to laziness. (conscientiousness_1_r)

German: Ich bin bequem, neige zur Faulheit.

1. I'm relaxed, I don't let stress upset me calm. (neuroticism_1_r)

German: Ich bin entspannt, lasse mich durch Stress nicht aus der Ruhe bringen.

1. I have little artistic interest. (openness_1_r)

German: Ich habe nur wenig künstlerisches Interesse.

1. I get out of myself, I'm sociable. (extraversion_2)

German: Ich gehe aus mir heraus, bin gesellig.

1. I tend to criticize others. (agreeableness_2_r)

German: Ich neige dazu, andere zu kritisieren.

1. I complete tasks thoroughly. (conscientiousness_2)

German: Ich erledige Aufgaben gründlich.

1. I get nervous and insecure easily. (neuroticism_2)

German: Ich werde leicht nervös und unsicher.

1. I have an active imagination, I am imaginative. (openness_2)

German: Ich habe eine aktive Vorstellungskraft, bin fantasievoll.

We included 5 items assessing typical phone use (based on Konok et al., 2016).

Instructions: Please indicate the extent to which the following statements are characteristic of you in terms of your typical phone use.

German: Bitte geben Sie an, wie charakteristisch die folgenden Aussagen für Ihren typischen Umgang mit Ihrem Smartphone sind.

Scoring: (1) not at all characteristic of me, (7) very characteristic of me

German: (1) überhaupt nicht charakteristisch für mich, (7) sehr charakteristisch für mich

- I regularly check my phone even if it does not ring. [checking_1]

German: Ich schaue regelmäßig auf mein Smartphone, auch wenn es nicht klingelt.

- I usually check push notifications I receive on my phone. [checking_2]

German: Normalerweise reagiere ich auf Push-Benachrichtigungen, die ich auf meinem Smartphone erhalte.

- My phone is within my reach all day long. [separation_1]

German: Mein Smartphone ist den ganzen Tag in meiner Reichweite.

- My phone is always charged. [separation_2]

German: Mein Smartphone ist immer aufgeladen.

- I never leave my phone at home when I go out. [separation_3]

German: Ich lasse mein Smartphone nie zu Hause, wenn ich rausgehe.

**Laboratory session and experience sampling**

The following measures were repeatedly used both in the laboratory session and during the experience sampling phase.

Momentary affective states will be assessed using items from a validated German measure specifically designed to reliably capture within-person variability (Wilhelm & Schoebi, 2007). The measure is based on the Multidimensional Mood Questionnaire (MDMQ; Steyer et al., 1997) that assesses basic diffuse affect dimensions. We will ask participants to indicate levels of arousal (ranging from calm to tense) and valence (ranging from pleasant to unpleasant) using each two bipolar items.

Scoring: Using a slider from the start position 0 to a maximum of 9.

At the moment, I feel …

German: Im Moment fühle ich mich …

- … relaxed-tense. (arousal_1)

German: entspannt-angespannt

- … agitated-calm. (arousal_2)

German: unruhig-ruhig

- … content-discontent. (valence_1)

German: zufrieden-unzufrieden

- … unwell-well. (valence_2)

German: unwohl-wohl

We assessed momentary dissociative experiences using four items from the German version of the Dissociative Symptoms Scale Brief Form (DSS-B; Macia et al., 2022; translation by Nikolaus Kleindienst, personal communication, April 04, 2023).

Scoring: Using a slider from the start position 0 (*not present*) to a maximum of 4 (*very strong*).

German: 0 (*überhaupt nicht*) 4 (*sehr stark*)

At the moment, …

German: Im Moment …

- … things around me seem strange or unreal. [depder_1]

German: wirken die Dinge um mich herum fremd oder unwirklich.

- … I feel like I am in a movie – like nothing that is happening is real. [depder_2]

German: fühle ich mich wie in einem Film – alles, was passiert, wirkt unwirklich.

- … I am not paying attention to what is going on around me. [gaps_1]

German: merke ich nicht, was um mich herum vorgeht.

- … I am so focused on something going on in my mind that I lose track of what is happening around me. [gaps_2]

German: bin ich so in meine Gedanken vertieft, dass ich nicht mitbekomme, was geschieht.

The following measures were repeatedly during the experience sampling phase to collect context information.

We assessed hourly situational experiences using each one item for three subscales of the German Personality Dynamics Diary (Zimmermann et al., 2019). We chose the items with the largest within-person factor loadings.

Scoring: 0 (*no*), 1 (*yes*)

German: 0 (*nein*), 1 (*ja*)

In the past hour ...

German: In der letzten Stunde ...

- ... I was ignored or rejected by others. [social_stress]

German: ... wurde ich von anderen ignoriert, abgelehnt oder zurückgewiesen.

- ... I had a good time with others (e.g., interesting or funny conversations). [positive_event]

German: … hatte ich eine gute Zeit mit anderen (z.B. interessante oder lustige Gespräche).

- … I was under high pressure to succeed while getting done with my tasks. [workload]

German: … stand ich beim Erledigen meiner Aufgaben unter großem Erfolgsdruck.

- … I exercised. [excercise]

German: … habe ich Sport gemacht.

The following measures were used at the start of each day during the experience sampling phase to collect additional context information.

Scoring: Using a slider from the start position 0 (*not at all*) to a maximum of 6 (*very*).

German: 0 (*überhaupt nicht*) 6 (*sehr*)

Last night …

Letzte Nacht …

- ... I had a problem with my sleep. [sleep_disturbance]

German: … hatte ich ein Problem mit meinem Schlaf.

The following measures were used at the end of each day during the experience sampling phase to collect additional context information.

Scoring: Using a slider from the start position 0 (*not at all*) to a maximum of 6 (*very*).

German: 0 (*überhaupt nicht*) 6 (*sehr*)

Today …

Heute …

- ... I felt stressed. [stress]

German: … fühlte ich mich gestresst.

- ... I felt fearful. [anxiety]

German: … fühlte ich mich ängstlich.

- ... I felt worthless. [depression]

German: … fühlte ich mich wertlos.

- ... I felt left out. [isolation]

German: … fühlte ich mich ausgegrenzt.

Scoring: 0 (*no*), 1 (*yes*)

German: 0 (*nein*), 1 (*ja*)

Today …

Heute…

- ... I have used legal or illegal drugs other than alcohol and prescribed medications (e.g., cannabis, ketamine). [day_drugs]

German: … habe ich legale oder illegale Drogen außer Alkohol, Nikotin, Koffein, verschriebene Dauermedikation konsumiert (z.B. Cannabis, Ketamin).

**References**

Carlson, E. B., Waelde, L. C., Palmieri, P. A., Macia, K. S., Smith, S. R., & McDade-Montez, E. (2018). Development and validation of the Dissociative Symptoms Scale. *Assessment*, *25*(1), 84–98. https://doi.org/10.1177/1073191116645904

Freyberger, H. J., Spitzer, C., Stieglitz, R. D., Kuhn, G., Magdeburg, N., & Bernstein-Carlson, E. (1998). Fragebogen zu dissoziativen Symptomen (FDS): Deutsche Adaptation, Reliabilität und Validität der amerikanischen Dissociative Experience Scale (DES). *Psychotherapie Psychosomatik Medizinische Psychologie*, *48*(6), 223–229.

Gutzweiler, R., & In-Albon, T. (2018) Überprüfung der Gütekriterien der deutschen Version der Difficulties in Emotion Regulation Scale in einer klinischen und einer Schülerstichprobe Jugendlicher. *Zeitschrift für Klinische Psychologie und Psychotherapie*, *47*(4), 274-286. https://doi.org/10.1026/1616-3443/a000506

Harlow, S. D., Gass, M., Hall, J. E., Lobo, R., Maki, P., Rebar, R. W., Sherman, S., Sluss, P. M., & de Villiers, T. J. (2013): Executive summary of the Stages of Reproductive Aging Workshop + 10: Addressing the unfinished agenda of staging reproductive aging. *Menopause*, *19*(4), 387-395. https://doi.10.1097/gme.0b013e31824d8f40

Kaufman, E. A., Xia, M., Fosco, G., Yaptangco, M., Skidmore, C. R., & Crowell, S. E. (2015). The Difficulties in Emotion Regulation Scale Short Form (DERS-SF): Validation and replication in adolescent and adult samples. Journal of Psychopathology and Behavioral *Assessment*, *38*(3), 443–455. https://doi.org/10.1007/s10862-015-9529-3

Kerber, A., Schultze, M., Müller, S., Rühling, R. M., Wright, A. G. C., Spitzer, C., Krueger, R. F., Knaevelsrud, C., & Zimmermann, J. (2022). Development of a short and ICD-11 compatible measure for DSM-5 maladaptive personality traits using ant colony optimization algorithms. *Assessment*, *29*(3), 467–487. https://doi.org/10.1177/1073191120971848

Klusmann, H., Schulze, L., Engel, S., Bücklein, E., Daehn, D., Lozza-Fiacco, S., Geiling, A., Meyer, C., Andersen, E., Knaevelsrud, C. & Schumacher, S. (2022). HPA axis activity across the menstrual cycle - a systematic review and meta-analysis of longitudinal studies. *Frontiers in Neuroendocrinology*, *66*, 900-998. https://doi.org/10.1016/j.yfrne.2022.100998

Konok, V., Gigler, D., Bereczky, B. M., & Miklósi, Á. (2016). Humans’ attachment to their mobile phones and its relationship with interpersonal attachment style. *Computers in Human Behavior*, *61*, 537–547. https://doi.org/10.1016/j.chb.2016.03.062

Kroenke, K., Strine, T., Spitzer, R. L., Williams, J., Berry, J., & Mokdad, A. H. (2008). The PHQ-8 as a measure of current depression in the general population. *Journal of Affective Disorders*, *114*(3). https://doi.org/10.1016/j.jad.2008.06.026

Löwe, B., Kroenke, K., Herzog, W., & Gräfe, K. (2004). Measuring depression outcome with a brief self-report instrument: sensitivity to change of the Patient Health Questionnaire (PHQ-9). *Journal of Affective Disorders*, *81*(1), 61–66. https://doi.org/10.1016/s0165-0327(03)00198-8

Linares, N. F., Charron, V., Ouimet, A. J., Labelle, P. R., & Plamondon, H. (2020). A systematic review of the Trier Social Stress Test methodology: Issues in promoting study comparison and replicable research. *Neurobiology of Stress*, *13*(5), 100–235. https://doi.org/10.1016/j.ynstr.2020.100235

Macia, K. S., Carlson, E. B., Palmieri, P. A., Smith, S. R., Anglin, D. M., Ghosh Ippen, C., … Waelde, L. C. (2022). Development of a brief version of the Dissociative Symptoms Scale and the reliability and validity of DSS-B scores in diverse clinical and community samples. *Assessment*, *107*(3),113–133. https://doi.org/10.1177/10731911221133317

Olderbak, S., Uusberg, A., MacCann, C., Pollak, K. M., & Gross, J. J. (2022). The Process Model of Emotion Regulation Questionnaire: Assessing individual differences in strategy stage and orientation. *Assessment*. Advance online publication. https://doi.org/10.1177/10731911221134601

Rammstedt, B., Kemper, C. J., Klein, M. C., Beierlein, C., & Kovaleva, A. (2017). A short scale for assessing the Big Five dimensions of personality: 10 item Big Five inventory (BFI-10). *Methoden, Daten, Analysen*, *7*(2), 233-249. https://doi.org/10.12758/mda.2013.013

Steyer, R., Schwenkmezger, P., Notz, P., & Eid, M. (1994). Testtheoretische Analysen des Mehrdimensionalen Befindlichkeitsfragebogen (MDBF) [Theoretical analysis of a multidimensional mood questionnaire (MDBF)]. *Diagnostica*, *40*(4), 320–328

Wilhelm, P., & Schoebi, D. (2007). Assessing mood in daily life. *European Journal of*

*Psychological Assessment*, *23*(4), 258–267. https://doi.org/10.1027/1015-5759.23.4.258

Wingenfeld, K., Spitzer, C., Mensebach, C., Grabe, H., Hill, A., Gast, U., Schlosser, N., Höpp,

H., Beblo, T., &amp; Driessen, M. (2010). Die Deutsche Version Des Childhood Trauma Questionnaire (CTQ): Erste Befunde zu den psychometrischen Kennwerten. *Psychotherapie, Psychosomatik, Medizinische Psychologie*, *60*(08). https://doi.org/10.1055/s-0030-1253494

Wittmann, L., Dimitrijevic, A., Ehlers, A., Foa, E. B., Kessler, H., Schellong, J., & Burgmer, M. (2021). Psychometric properties and validity of the German version of the Post-Traumatic Diagnostic Scale for DSM-5 (PDS-5). *European Journal of Psychotraumatology*, *12*(1). https://doi.org/10.1080/20008198.2021.1965339

Zimmermann, J., Woods, W. C., Ritter, S., Happel, M., Masuhr, O., Jaeger, U., Spitzer, C., & Wright, A. G. C. (2019). Integrating structure and dynamics in personality assessment: First steps toward the development and validation of a personality dynamics diary. *Psychological Assessment*, *31*(4), 516–531. https://doi.org/10.1037/pas0000625
