# Supplementary material for: The temporal dynamics of dissociation: protocol for an ecological momentary assessment and laboratory study in a transdiagnostic sample
Source: BMC Psychol. 2023 Jun 7;11:178. doi: 10.1186/s40359-023-01209-z (PMC10245627; doi:10.1186/s40359-023-01209-z)
Supplement: Supplementary file 2 — Additional file 2. Clinician administered interviews. [file 40359_2023_1209_MOESM2_ESM.docx]

This document contains a list of all clinical interviews used in the project. The interviews will be administered by psychologists holding a master’s degree (or equivalent) and physicians with specialized training in psychiatry and psychotherapy.

**Clinician Administered Interviews**

The following interviews were included to screen participants for eligibility (inclusion criteria).

We will administer the PTSD section of the German version of the *Structured Clinical Interview for DSM-5 Disorders – Clinical Version* (SCID-CV; Beesdo-Baum et al., 2019a) to determine presence or absence of a PTSD diagnosis in the past month.

We will administer the BPD section of the German version of the *Structured Clinical Interview for DSM-5 Disorders – Personality Disorders* (SCID-PD; Beesdo-Baum et al., 2019b) to determine presence or absence of a BPD diagnosis.

We will administer the *Structured Clinical Interview for Dissociative Symptoms and Disorders* (Steinberg, 2023) to determine presence or absence of a dissociative disorder diagnosis.

The following interviews were included to screen participants for eligibility (exclusion criteria).

We will administer relevant sections of the German version of the *Structured Clinical Interview for DSM-5 Disorders – Clinical Version* (SCID-CV; Beesdo-Baum et al., 2019) to determine presence or absence of current (severe) major depressive disorder, lifetime bipolar disorder, any lifetime psychotic disorder, substance use disorders, and eating disorders (especially anorexia nervosa). We will also determine presence or absence of other non-exclusionary comorbid diagnoses such as anxiety disorders and obsessive-compulsive disorder.

**References**

Beesdo-Baum, K., Zaudig, M., & Wittchen, H. (2019a). *Strukturiertes Klinisches Interview für DSM-5: Klinische Version [Structured Clinical Interview for DSM-5-Disorders: Clinical Version]*. Hogrefe.

Beesdo-Baum, K., Zaudig, M., & Wittchen, H. (2019b). *Strukturiertes Klinisches Interview für DSM-5: Persönlichkeitsstörungen [Structured Clinical Interview for DSM-5-Disorders: Personality Disorders]*. Hogrefe.

Steinberg, M. (2023). *The SCID-D interview the SCID-D interview: Dissociation assessment in therapy, forensics, and research*. American Psychiatric Association Publishing.
