# Supplementary material for: The temporal dynamics of dissociation: protocol for an ecological momentary assessment and laboratory study in a transdiagnostic sample
Source: BMC Psychol. 2023 Jun 7;11:178. doi: 10.1186/s40359-023-01209-z (PMC10245627; doi:10.1186/s40359-023-01209-z)
Supplement: Supplementary file 3 — Additional file 3. Laboratory session. [file 40359_2023_1209_MOESM3_ESM.docx]

This document contains instructions for the laboratory session and stress induction used in the project.

Participants will have at least 60 minutes resting time upon arriving at the laboratory before the laboratory session begins to stabilize physiological responses, which may vary across participants. Participants are asked to abstain from brushing or flossing their teeth, smoking, using substances, drinking alcohol or caffeinated beverages, engaging in physical exercise, and eating during this time.

The stress induction will be administered within the period of 1:00 PM to 3:00 PM.

A protocol for the laboratory session is available at https://www.protocols.io/view/tsst-cukywuxw (ID: 75538).

**Vor dem TSST (Raum A)**

Vor dem TSST zeigt und erklärt der Versuchsleiter dem Probanden die drei mobilen Geräte zur kontinuierlichen Erfassung von Herzrate (Vivalink EKG Patch) und Blutdruck (iHealth BP Cuff) und Hautleitwiderstand (Empatica Smartwatch). Die drei Geräte werden vom Probanden anschließend selbst angelegt.

Der Versuchsleiter verbindet die Geräte über die Vivalink App (Multi Vital Monitor) und Empatica App. Der Versuchsleiter stell sicher, dass die kontinuierlichen Messungen von Herzrate und Hautleitwiderstand begonnen haben.

Danach wird dem Probanden erklärt, dass während der Laboruntersuchung Speichelproben genommen werden, die zur Messung des Cortisolspiegels verwendet werden. Außerdem wird der Blutdruck wiederholt über die Manschette von iHealth gemessen.

Danach lädt der Versuchsleiter den Probanden über das m-Path Dashboard zur TSST-Befragung ein („tsst prompt“ hinzufügen, dann „send to client“). Dann beantwortet der Proband die Fragen ein erstes Mal auf dem eigenen Handy. Der Proband wird informiert, dass die Richtigkeit der Angaben sehr wichtig ist für das Forschungsvorhaben und dass der Proband die Fragen so beantworten soll, wie im aktuellen Moment die Fragen so beantwortet werden sollen, wie es in diesem Moment ist. *[Messung -10]*

Der Versuchsleiter instruiert den Probanden die erste Speichelprobe zu entnehmen (Salivette Cortisol für 2 Minuten in Mund legen, nicht kauen). Währenddessen erste Blutdruckmessung im Sitzen am nichtdominanten Arm. *[Messung -10]*

Anschließend erklärt der Versuchsleiter, dass es für die Laboruntersuchung wichtig ist, dass keine Mittel zur Anspannungs- und Emotionsregulation (z.B. Stressball, Ammoniak, usw.) verwendet werden. Der Versuchsleiter bittet den Probanden die Mittel zu übergeben und sagt, dass er die Mittel für die Zeit der Laboruntersuchung verwahren wird.

Falls der Proband danach fragt, sagt der Versuchsleiter, dass die Mittel selbstverständlich bei dringendem Bedarf sofort zur Verfügung gestellt werden. Um dies sicherzustellen werden die Mittel in einer verschlossenen undurchsichtigen Box im Raum B aufbewahrt. Die Box befindet sich außerhalb des Sichtfelds des Probanden.

Nach der Wartezeit fordert der Versuchsleiter den Probanden auf, über die m-Path App die Fragen ein weiteres Mal zu beantworten. Anschließend wird die zweite Speichelprobe entnommen und erneut der Blutdruck gemessen. *[Messung 0]*

Der Versuchsleiter bringt den Probanden in den Raum B, in dem das Komitee bereits sitzt.

**Während des TSST (Raum B)**

Verhalten des Auswahlkomitees (aktiver und passiver Stressor, ein Mann und eine Frau) während der Einführung durch den Versuchsleiter:

- ernste Stimmung aufrechterhalten
- nicht sprechen, lachen o.ä.
- evtl. den Gruß der VP mit einem knappen „Guten Tag“ beantworten
- Augenkontakt nicht vermeiden

Das Auswahlkomitee trägt weiße, zugeknöpfte Kittel. Kein sichtbares Namensschild.

TSST-Instruktion für den Versuchsleiter:

Ihre Aufgabe besteht nun darin, vor diesem Auswahlkomitee eine Rede für eine Bewerbung zu halten.

Sie haben gleich 5 Minuten Zeit, um sich hier auf diese Rede vorzubereiten. Sie können sich dazu Notizen machen, dürfen diese aber nicht während der Rede benutzen.

Stellen Sie sich vor, dass Sie sich für eine Stelle, die zu Ihrem bisherigen Werdegang passt, beworben haben und dass Sie dort zum Vorstellungsgespräch eingeladen wurden oder dass Sie sich für eine Beförderung bewerben, die für Sie mehr Geld, mehr Verantwortung und interessantere Aufgaben bedeutet.

*Hinweise: Bei Studierenden entsprechend der Qualifikation oder entsprechend des Ziels der Ausbildung.*

Von Ihnen wird erwartet, dass Sie eine freie Rede halten. Es wird verlangt, dass Sie dem Komitee in fünf Minuten erklären, warum Sie glauben, die beste Kandidatin für diese Stelle zu sein. Dazu stellen Sie sich nach Ihrer Vorbereitungszeit hinter das Mikrofon, so dass Ihre Stimme aufgenommen wird. Beachten Sie, dass Sie von der Kamera ebenfalls aufgenommen werden. Die Geräte dienen späteren Stimm- und Verhaltensanalysen.

Die Komiteemitglieder sind in Verhaltensbeobachtung geschult und werden sich während der Rede Notizen machen. Lassen Sie sich dadurch nicht beunruhigen. Sie sollten versuchen, den bestmöglichen Eindruck zu hinterlassen und die Rolle des Bewerbers während der Rededauer so gut wie möglich zu verkörpern. Dem Komitee ist das Recht vorbehalten, falls Unklarheiten auftreten, weitere Fragen zu stellen.

Nach Ihrer Rede, die fünf Minuten dauern soll, wird Ihnen das Komitee eine zweite Aufgabe stellen, die auch vom Komitee erläutert wird. Diese wird wieder fünf Minuten dauern. Haben Sie noch irgendwelche Fragen?

Der Versuchsleiter und das Komitee verlassen den Raum. Ca. 5 Minuten Vorbereitungszeit.

Nach der Vorbereitungszeit:

Der Proband beantwortet erneut die Fragen zu Ihrem aktuellen Befinden über die m-Path App. Außerdem wird eine Speichelprobe entnommen. *[Messung +10]*

Aktiver Stressor: **„Ihre Vorbereitungszeit ist vorbei. Kommen Sie nun nach vorne. (Pause) Stellen Sie sich hinter das Mikrofon auf die Bodenmarkierung (Kreuz) und beginnen Sie mit Ihrem Vortrag, wenn die Aufnahmegeräte eingeschaltet sind.“**

Passiver Stressor: Einschalten der Kamera / des Rekorders, Stoppuhr starten

Aktiver Stressor: **„Bitte beginnen Sie jetzt mit Ihrem Vortrag.**

Wichtig: Ziel des TSST ist es, eine Stressreaktion hervorzurufen, nicht Ärger. Das Komitee sollte alles unterlassen, was den Probanden verärgern könnte**.**

Während der ersten TSST-Aufgabe (Vortrag):

- Augenkontakt mit VP suchen und halten
- Abwechselndes Notizenmachen und Aufschauen zur VP
- Mindestredezeit 3 Minuten
- **Bei erster Pause der VP 20 (!) Sekunden schweigen**
- Bei Schweigen länger als 20 Sekunden:

Aktiver Stressor: **„Sie haben noch Zeit. Fahren Sie bitte fort.“**

Aktiver Stressor: **„Ihre Vortragszeit ist noch nicht vorbei.“**

- Einwände ignorieren und wiederholt freundlich zum Weiterreden auffordern.
- Auf Fragen der VP nicht eingehen, sondern auf die Aufgabe hinweisen und dass alles Weitere nach dem Versuch geklärt wird.
- Die VP so lange wie möglich frei über seine Eigenschaften sprechen lassen.
- Darauf achten, dass die VP auch über ihre Persönlichkeit spricht, nicht nur fachliche Qualifikationen oder ähnliches darbietet. Eventuell eingreifen:

Aktiver Stressor: **„Ihr Lebenslauf/ Ihre Zeugnisse liegen uns bereits vor, bitte berichten Sie weiter über Ihre persönlichen Eigenschaften.“**

Aktiver Stressor: **„Kommen Sie noch auf Ihre negativen Eigenschaften zu sprechen“**

- Ab 3 Minuten Fragen mit Bezug auf die Vorstellungssituation und nach 20 Sekunden Schweigen:

„**Weshalb glauben Sie, dass gerade Sie für diese … besonders geeignet sind?“**

**„Weshalb sind Sie geeigneter als andere Bewerber?“**

**„Welche Eigenschaften von Ihnen machen Sie eher für den Job ungeeignet?“**

**„Welche Führungsqualitäten besitzen Sie?“**

**„Beschreiben Sie uns andere … , die Sie schon einmal ausgeübt haben, bzw. die Sie ausüben könnten.“**

**„Was halten Sie von Teamarbeit?“**

**„Wo sehen Sie Ihre Position in einem Team?“**

**„Denken Sie einmal an Ihren letzten Misserfolg. Wie sind Sie damit umgegangen?“**

- Wenn auch damit Probleme hat, stellt das Komitee allgemeine Fragen:

**„Erläutern Sie uns, inwiefern Ihre Hobbies in Bezug stehen zu … .“**

**„Welche anderen Interessen haben Sie?“**

**„Beschreiben Sie uns einen normalen Tagesablauf in Ihrem Leben.“**

**„Erklären Sie, weshalb es für Sie wichtig ist, Leistung zu erbringen.“**

**„Was halten Sie von der Aussage: ,Ein Spatz in der Hand ist besser als die Taube auf dem Dach’?“**

**„Halten Sie sich für einen Menschen, dem seine Unabhängigkeit wichtig ist?“**

**„Erachten Sie es für wichtig, dass man sich ein Leben lang weiterentwickelt?“**

**„Was halten Sie von Freunden?“**

Vor der zweiten TSST-Aufgabe (Rechnen):

Aktiver Stressor: **„Danke - das soll hier erst einmal genügen. Wir werden Ihnen gleich die zweite Aufgabe erläutern.“**

Versuchsleiter betritt Raum und fordert den Probanden auf, über die m-Path App die Fragen ein weiteres Mal zu beantworten. Anschließend wird eine Speichelprobe entnommen und erneut der Blutdruck gemessen. *[Messung +15]*

Während der zweiten TSST-Aufgabe (Rechnen):

Aktiver Stressor: **„Wir werden Ihnen nun die zweite Aufgabe erläutern. Es handelt sich hierbei um eine Rechenaufgabe. Sie sollen so schnell und korrekt wie möglich in 13er Schritten rückwärts bis Null rechnen. Sie fangen an bei 1022. Sollten Sie sich verrechnen, so weisen wir Sie darauf hin und Sie beginnen noch mal bei 1022. Haben Sie noch Fragen?**

**Dann beginnen Sie bitte.“**

- Passiver Stressor startet Stoppuhr erneut.
- Wenn sich der Proband sich verrechnet:

Aktiver Stressor: **„Stopp. 1022.“**

Aktiver Stressor: **„Fehler. 1022.“**

- Wenn der Proband längere Zeit nicht weiterkommt:

Aktiver Stressor: **„Fangen Sie noch einmal bei 1022 an.“**

- Wenn der Proband leise spricht:

Aktiver Stressor: **„Bitte sprechen Sie lauter!“** (auf Nachfrage, sagen, dass lautes Sprechen für eine gute Tischmikrofonaufzeichnung nötig sei)

- Wenn der Proband an die Decke oder auf den Boden schaut:

Aktiver Stressor: **„Schauen Sie bitte in die Kamera!“**

- Bei starken Rechenschwierigkeiten: Verstärkung durch bejahendes Nicken.

Falls dann noch Schwierigkeiten: Vereinfachung

„Wir verändern die Aufgabe ein wenig. Rechnen Sie nun in 7er Schritten rückwärts. Fangen Sie wieder bei 1022 an.“

- Zur Not 7er addieren lassen
- Einwände nicht gelten lassen und auf die noch nicht abgelaufene Zeit hinweisen.
- Nach Ablauf der zweiten 5 Minuten TSST beenden

**„Vielen Dank. Das Vorstellungsgespräch ist nun vorbei. Nehmen Sie bitte Platz und warten Sie auf den Versuchsleiter.“**

- Geräte demonstrativ ausschalten
- Das Gremium verlässt den Raum und der Versuchsleiter kehrt zurück

Versuchsleiter betritt Raum und fordert den Probanden auf, über die m-Path App die Fragen ein weiteres Mal zu beantworten. Anschließend wird eine Speichelprobe entnommen und erneut der Blutdruck gemessen. *[Messung +20]*

**Nach dem TSST (Raum A)**

Der Versuchsleiter bringt den Probanden zurück in Raum A.

10, 20 und 30 Minuten nach der vorangegangenen Messung fordert der Versuchsleiter den Probanden auf, über die m-Path App die Fragen ein weiteres Mal zu beantworten. Nach jeder Befragung wird eine Speichelprobe entnommen und erneut der Blutdruck gemessen. *[Messungen +30 +40 +50]*

Das Debriefing findet nach der allerletzten Messung statt. Der Versuchsleiter erklärt, dass das Prozedere, an dem der Proband teilgenommen hat, als Trier Social Stress Test bekannt ist. Es handelt sich um ein Verfahren der experimentellen Psychologie, bei dem im Labor moderater psychosozialer Stress ausgelöst und eine physiologische Stressreaktion hervorgerufen wird. Es wurden in Wirklichkeit keine Video- und Audioaufzeichnungen gemacht. Nach Möglichkeit kommen der aktive und der passive Stressor zum Debriefing dazu und geben sich freundlich als wissenschaftliches Personal zu erkennen.

**Anhang**

| 1022 - 13 | | |
| --- | --- | --- |
| 1022 |  |  |
| 1009 | 710 | 411 |
| 996 | 697 | 398 |
| 983 | 684 | 385 |
| 970 | 671 | 242 |
| 957 | 658 | 229 |
| 944 | 645 | 216 |
| 931 | 632 | 203 |
| 918 | 619 | 190 |
| 905 | 606 | 177 |
| 892 | 593 | 164 |
| 879 | 580 | 151 |
| 866 | 567 | 138 |
| 853 | 554 | 125 |
| 840 | 541 | 112 |
| 827 | 528 | 99 |
| 814 | 515 | 86 |
| 801 | 502 | 73 |
| 788 | 489 | 60 |
| 775 | 476 | 47 |
| 762 | 463 | 34 |
| 749 | 450 | 21 |
| 736 | 437 | 8 |
| 723 | 424 | 0 |

| 1022 - 7 | | | |
| --- | --- | --- | --- |
| 1022 |  |  |  |
| 1015 | 749 | 483 | 217 |
| 1008 | 742 | 476 | 210 |
| 1001 | 735 | 469 | 203 |
| 994 | 728 | 462 | 196 |
| 987 | 721 | 455 | 189 |
| 980 | 714 | 448 | 182 |
| 973 | 707 | 441 | 175 |
| 966 | 700 | 434 | 168 |
| 959 | 693 | 427 | 161 |
| 952 | 686 | 420 | 154 |
| 945 | 679 | 413 | 147 |
| 938 | 672 | 406 | 140 |
| 931 | 665 | 399 | 133 |
| 924 | 658 | 392 | 126 |
| 917 | 651 | 385 | 119 |
| 910 | 644 | 378 | 112 |
| 903 | 637 | 371 | 105 |
| 896 | 630 | 364 | 98 |
| 889 | 623 | 357 | 91 |
| 882 | 616 | 350 | 84 |
| 875 | 609 | 343 | 77 |
| 868 | 602 | 336 | 70 |
| 861 | 595 | 329 | 63 |
| 854 | 588 | 322 | 56 |
| 847 | 581 | 315 | 49 |
| 840 | 574 | 308 | 42 |
| 833 | 567 | 301 | 35 |
| 826 | 560 | 294 | 28 |
| 819 | 553 | 287 | 21 |
| 812 | 546 | 280 | 14 |
| 805 | 539 | 273 | 7 |
| 798 | 532 | 266 | 0 |
| 791 | 525 | 259 |  |
| 784 | 518 | 252 |  |
| 777 | 511 | 245 |  |
| 770 | 504 | 238 |  |
| 763 | 497 | 231 |  |
| 756 | 490 | 224 |  |
