# Supplementary material for: The temporal dynamics of dissociation: protocol for an ecological momentary assessment and laboratory study in a transdiagnostic sample
Source: BMC Psychol. 2023 Jun 7;11:178. doi: 10.1186/s40359-023-01209-z (PMC10245627; doi:10.1186/s40359-023-01209-z)
Supplement: Supplementary file 4 — Additional file 4. Dissociative states measure. [file 40359_2023_1209_MOESM4_ESM.docx]

This document contains information on the selection of dissociative phenomena studied in the current project, as well as information on scale development.

**Limitations of Current Measures of State Dissociation**

To our best knowledge, no validated dissociative states measure for the use in dense experience sampling studies is available. The 21-items Dissociation Tension Scale Acute (DSS-acute; Stiglmayr et al., 2003 has been used in German speaking samples. A 4-items version of the scale was developed for the use before and after neuropsychological sessions and has demonstrated within-person variability (DSS-4; Stiglmayr et al., 2009). The authors recommend using the DSS-4 in experience sampling studies. However, there are three reasons why we decided not to use the DSS-4 in this study. First, although the authors suggested that the DSS-acute assesses different dissociative experiences (e.g., psychological vs. somatoform), initial evidence did not support the existence of any sub-factors and the authors recommended the use of a single dissociative factor (Stiglmayr et al., 2003), which conflicts with the widely held definition of dissociation as a multifaceted construct and available evidence (e.g., American Psychiatric Association, 2022; Carlson et al., 2018). Second, the selection of the items for the DSS-4 was based on the judgement of the authors rather than on evidence, which raises concerns about content validity (e.g., it remains unclear why the scale includes an item on analgesia but not gaps in awareness/memory). Third, the authors report no estimates for within-person reliability for the DSS-4, although demonstrating sufficient within-person reliability is crucial for any experience sampling scale (Brose et al., 2022). In fact, there is some evidence suggesting that within-person reliability of the DSS-4 may be poor. For example, results from one laboratory study that administered the DSS-4 before and after a stress induction in patients with BPD and/or PTSD fail to support measurement invariance of a single dissociation factor based on the DSS-4 item scores, and rather suggest that the DSS-4 assesses two or more qualitatively different dissociation constructs (Graumann et al., 2023). Maybe because the DSS-4 does not assess a single-faceted construct (which, if true, would show in low within-person reliability estimates), experience sampling studies that applied the DSS-4 only used the depersonalization/derealization items, dropping or replacing the analgesia and somatoform dissociation items (e.g., Heekerens et al., 2023; Soffer-Dudek et al., 2017; Vannikov-Lugassi et al., 2018). One study found acceptable within-person reliability for the depersonalization/derealization items (McDonald’s omega = .66; Heekerens et al., 2023).

**Advantages of the Dissociation Symptoms Scale**

Because of the limitations of the DSS-4 scale, we looked for alternatives and after carefully reviewing the literature decided to use the Dissociative Symptoms Scale (DSS; Carlson et al., 2018). The DSS has been developed to capture levels of moderate pathological dissociative experiences, which aligns well with the aim of the current project. It is available as an 8-items short form (DSS-B; Macia et al., 2022) and has been translated to German by Nikolaus Kleindienst (personal communication, April 04, 2023). The DSS was developed along the lines of a theoretically sound concept of dissociative and related symptoms that differentiates depersonalization/derealization, sensory misperceptions, cognitive/behavioral reexperience, and gaps in awareness/memory – and these sub-factors are supported by available evidence in patients with PTSD (Carlson et al., 2018). The items for the short form were selected based on empirical criteria, especially the authors selected the two items that contributed most to their respective sub-factor (Maci et al., 2020). More so, a subset of 10 items of the DSS, out of which 4 items overlap with the DSS-B, has demonstrated within-person variability in an experience sampling study that used retrospective assessments over the past 4 hours in patients with PTSD (Carlson et al., 2016). For this study, we adapted the time scale of the items to assess experiences in the moments rather than retrospective experiences (Carlson et al., 2016). We chose to assess depersonalization/derealization and gaps in awareness/memory (amnesia) because those experiences are at the core of the DSM-5 definition of dissociation (American Psychiatric Association, 2022).­­­

| **No** | **DSS-B Item** | **German translation** | **State adaptation** | **German translation** |
| --- | --- | --- | --- | --- |
|  | Subscale: depersonalization/derealization | | | |
| 1 | Things around me seemed strange or unreal. | Die Dinge um mich herum wirkten fremd oder unwirklich. | At the moment, things around me seem strange or unreal. | Im Moment wirken die Dinge um mich herum fremd oder unwirklich. |
| 4 | I felt like I was in a movie – like nothing that was happening was real. | Ich fühlte mich wie in einem Film – alles was passierte, wirkte unwirklich. | At the moment, I feel like I am in a movie – like nothing that is happening is real. | Im Moment fühle ich mich wie in einem Film – alles, was passiert, wirkt unwirklich. |
|  | Subscale: gaps in awareness/memory | | | |
| 6 | I suddenly realized that I hadn’t been paying attention to what was going on around me. | Mir fiel plötzlich auf, dass ich nicht bemerkt hatte, was um mich herum vorging. | At the moment, I am not paying attention to what is going on around me. | Im Moment merke ich nicht, was um mich herum vorgeht. |
| 8 | I got so focused on something going on in my mind that I lost track of what was happening around me. | Ich war so in meine Gedanken vertieft, dass ich nicht mitbekam, was um mich herum vor sich ging. | At the moment, I am so focused on something going on in my mind that I lose track of what is happening around me. | Im Moment bin ich so in meine Gedanken vertieft, dass ich nicht mitbekomme, was um mich herum geschieht. |

*Note.* The original English items are taken from Macia et al. (2022). The German translation comes from Nikolaus Kleindienst (personal communication, April 04, 2023). The state adaptation comes from Johannes Heekerens. No = item number; DSS-B = Dissociative Symptoms Scale – Brief Form.

**References**

American Psychiatric Association (2022). *Diagnostic and statistical manual of mental disorders: DSM-5-TR*. American Psychiatric Publishing.

Brose, A., Schmiedek, F., Gerstorf, D., & Voelkle, M. C. (2020). The measurement of within-

person affect variation. *Emotion*, *20*(4), 677–699. https://doi.org/10.1037/emo0000583

Carlson, E. B., Field, N. P., Ruzek, J. I., Bryant, R. A., Dalenberg, C. J., Keane, T. M., & Spain, D. A. (2016). Advantages and psychometric validation of proximal intensive assessments of patient-reported outcomes collected in daily life. *Quality of Life Research: An International Journal of Quality of Life Aspects of Treatment, Care and Rehabilitation*, *25*(3), 507–516. https://doi.org/10.1007/s11136-015-1170-9

Carlson, E. B., Waelde, L. C., Palmieri, P. A., Macia, K. S., Smith, S. R., & McDade-Montez, E. (2018). Development and validation of the Dissociative Symptoms Scale. *Assessment*, *25*(1), 84–98. https://doi.org/10.1177/1073191116645904

Graumann, L., Heekerens, J. B., Duesenberg, M., Metz, S., Otte, C., Roepke, S., &

Wingenfeld, K. (2023). Association between baseline dissociation levels and stress-induced state dissociation in patients with posttraumatic-stress disorder, borderline personality disorder, and major depressive disorder. *Borderline Personality Disorder and Emotion Dysregulation*, *10*(1), 11. https://doi.org/10.1186/s40479-023-00215-2

Heekerens, J. B., Schulze, L., Enge, J., Renneberg, B., & Roepke, S. (2023). Affective arousal

temporally precedes dissociation in patients with borderline personality disorder: A preliminary experience sampling study. *Psychological Trauma: Theory, Research, Practice, and Policy*. Advance online publication. https://doi.org/10.1037/tra0001516

Macia, K. S., Carlson, E. B., Palmieri, P. A., Smith, S. R., Anglin, D. M., Ghosh Ippen, C., … Waelde, L. C. (2022). Development of a brief version of the Dissociative Symptoms Scale and the reliability and validity of DSS-B scores in diverse clinical and community samples. *Assessment*, 107(3),113–133. https://doi.org/10.1177/10731911221133317

Soffer-Dudek, N., Shelef, L., Oz, I., Levkovsky, A., Erlich, I., & Gordon, S. (2017). Absorbed

in sleep: Dissociative absorption as a predictor of sleepiness following sleep deprivation in two high-functioning samples. *Consciousness and Cognition*, *48*, 161–170. https://doi.org/10.1016/j.concog.2016.11.009

Stiglmayr, C. E., Braakmann, D., Haaf, B., Stieglitz, R.‑D., & Bohus, M. (2003). Entwicklung

und psychometrische Charakteristika der Dissoziations-Spannungs-Skala akut (DSS-

akut). *Psychotherapie, Psychosomatik und medizinische Psychologie*, *53*(7), 287–294.

https://doi.org/10.1055/s-2003-40495

Stiglmayr, C., Schmahl, C., Bremner, J. D., Bohus, M., & Ebner-Priemer, U. (2009).

Development and psychometric characteristics of the DSS-4 as a short instrument to assess dissociative experience during neuropsychological experiments. *Psychopathology*, 42(6), 370–374. https://doi.org/10.1159/000236908

Vannikov-Lugassi, M., & Soffer-Dudek, N. (2018). No time like the present: Thinking about

the past and the future is related to state dissociation among individuals with high levels of psychopathological symptoms. *Frontiers in Psychology*, *9*, 2465. doi:10.3389/fpsyg.2018.02465
